# A Movement Monitor Based on Magneto-Inertial Sensors for Non-Ambulant Patients with Duchenne Muscular Dystrophy: A Pilot Study in Controlled Environment

**DOI:** 10.1371/journal.pone.0156696

**Published:** 2016-06-07

**Authors:** Anne-Gaëlle Le Moing, Andreea Mihaela Seferian, Amélie Moraux, Mélanie Annoussamy, Eric Dorveaux, Erwan Gasnier, Jean-Yves Hogrel, Thomas Voit, David Vissière, Laurent Servais

**Affiliations:** 1 Institut de Myologie, Groupe Hospitalier de La Pitié Salpêtrière, Paris, France; 2 Service de Neurologie Pédiatrique, Groupe de Recherches sur l'Analyse Multimodale de la Fonction Cérébrale, Centre Hospitalier Universitaire Amiens—Picardie, Amiens, France; 3 SYSNAV, Vernon, France; University of Colorado Anschutz Medical Campus, UNITED STATES

## Abstract

Measurement of muscle strength and activity of upper limbs of non-ambulant patients with neuromuscular diseases is a major challenge. ActiMyo^®^ is an innovative device that uses magneto-inertial sensors to record angular velocities and linear accelerations that can be used over long periods of time in the home environment. The device was designed to insure long-term stability and good signal to noise ratio, even for very weak movements. In order to determine relevant and pertinent clinical variables with potential for use as outcome measures in clinical trials or to guide therapy decisions, we performed a pilot study in non-ambulant neuromuscular patients. We report here data from seven Duchenne Muscular Dystrophy (DMD) patients (mean age 18.5 ± 5.5 years) collected in a clinical setting. Patients were assessed while wearing the device during performance of validated tasks (MoviPlate, Box and Block test and Minnesota test) and tasks mimicking daily living. The ActiMyo^®^ sensors were placed on the wrists during all the tests. Software designed for use with the device computed several variables to qualify and quantify muscular activity in the non-ambulant subjects. Four variables representative of upper limb activity were studied: the rotation rate, the ratio of the vertical component in the overall acceleration, the hand elevation rate, and an estimate of the power of the upper limb. The correlations between clinical data and physical activity and the ActiMyo^®^ movement parameters were analyzed. The mean of the rotation rate and mean of the elevation rate appeared promising since these variables had the best reliability scores and correlations with task scores. Parameters could be computed even in a patient with a Brooke functional score of 6. The variables chosen are good candidates as potential outcome measures in non-ambulant patients with Duchenne Muscular Dystrophy and use of the ActiMyo^®^ is currently being explored in home environment.

***Trial Registration*:** ClinicalTrials.gov NCT01611597

## Introduction

The measurement and quantification of the autonomy of patients suffering from chronic illness requires the development of monitoring tools. To improve monitoring of disabilities and dependency caused by a disease, new e-health applications aim at transforming medical care practice for patients. Among the many aspects of e-health developments are those related to the acquisition of remote medical measures. The need to monitor patients in their daily life has generated a need for precision sensors that meet the constraints of use outside a clinical institution [1]. In a few fields of medicine, such as cardiology and epileptology, ambulatory monitoring devices have been used in clinical trials and practice for years [2,3].

Actimetry is often used to measure the activity of a subject over a period of time. It can be achieved through different approaches (combined or not) such as measurement of accelerations or angular velocities, typically within a three-dimensional space. Actimetry using accelerometry has been used as a physical activity assessment that is clinically relevant in able-bodied children [4], to quantitatively assess physical activity of patients with cerebral palsy [5,6], upper limb motor function and walking in patients with multiple sclerosis [7,8], and to detect seizures [9–12]. Gyroscopic systems have been used for localization [13] to analyze postural parameters involved in pathological gait in patients with hemiplegia and Parkinson disease [14], and to determine biomechanical and rotational parameters for orthopedic diseases [15].

Accelerometers and gyroscopes can be coupled within an inertial device to estimate the orientation of the device. These devices have been used to assess postural disorders [16], pathological gait [17], tremor in patients with Parkinson’s disease [18] or to quantify spasticity of patients suffering from stroke [19]. For Duchenne muscular dystrophy (DMD), potential applications include the assessment of gait and trunk movements [20] and monitoring patients’ the daily life [21]. Inertial systems may be further coupled with magnetometers to allow much more precise estimation of velocity and position [22]. Recently devices combining inertial and magnetic measurements have been applied to medical fields such as epileptology [23], rehabilitation [24] and autism care [25]. However, the medical applications are still restricted and continuous home monitoring has yet to be solidly implemented using this technology.

Duchenne muscular dystrophy is one of the most common neuromuscular diseases; it is caused by mutations in the gene encoding the dystrophin protein [26–28]. The wasting of skeletal muscle compromises the patient’s mobility, their physical activity, and quality of life [29] and leads to complete paralysis and premature death [30,31]. The recent results from new pharmaco-gene therapies are promising for DMD patients [32–35]. Significant motor improvement has been reported for ambulant patients treated to restore dystrophin function [33,36]. Other molecules are in clinical trials for ambulant and non-ambulant DMD patients (Clinical Trials Identifier NCT01254019, NCT01540409, and NCT01826474). Since the balance between clinical benefit and side effects will drive regulatory approval, social security reimbursement, and patient compliance, close follow-up of patients’ activity at home who participate in clinical trials or who are treated with approved therapies is thus of critical importance for both ambulant and non-ambulant patients, especially in the context of very expensive new medications or post-marketing approval studies.

The gold standard for DMD patient evaluation during trials is currently the 6-minute walk test [37]. This test reflects ambulant patients’ peak performance in a clinical setting. As this is a test dedicated to ambulant subjects, non-ambulant patients are prevented from participation in most clinical trials. In the rare trials open to the non-ambulant population, upper limb function is not yet considered as a primary outcome (ClinicalTrials.gov Identifier: NCT01027884, NCT01009294). The present assessment tools for upper limb function in non-ambulant DMD patients include observer-rated performance in a controlled environment and self-reported questionnaires [38–42]. Recently, collaborative efforts among medical doctors, physiotherapists, and patients have led to development of a novel scale for assessing performance of upper limbs [43], a stereo camera-based reachable workspace analysis system [44,45] or a skeletal tracking system [46]. These different approaches aim to quantify peak patient performance in a controlled environment. They all require patients to travel to the hospital and are subject to patient fatigue and motivation at a particular period in time [47]. A device that could measure patient activity at home would be valuable for clinical evaluations of therapies and for guiding treatment decisions, especially in non-ambulant patients. Indeed, the non-ambulant population would benefit significantly from such a device, since (i) no gold standard exists for non-ambulant patients, (ii) social and environmental factors probably influence upper limb activity less than ambulation does, and (iii) non-ambulant patient trips to investigation centers are more complicated than for ambulant patients making home-based assessment optimal. A major issue in the development of devices to quantify movement in non-ambulant patients is that the movements are often of very low amplitude, speed and acceleration; it therefore requires excellent and well-calibrated sensors to reach good signal to noise ratio, and good accuracy and integrity even for slow motion (a couple of degrees per second). Moreover, in a home environment, methods have to be applied which account for the caregivers or the wheelchair induced movements of the patient.

Our present study aimed to highlight the feasibility of quantifying the range of upper limb movements produced by non-ambulant patients, using magneto-inertial sensors. We developed a wireless movement monitor—ActiMyo^®^—which contains a three-axis accelerometer, a three-axis gyroscope, and a three-axis magnetometer. This new tool is light and easy to wear and to use at home and during the patient’s daily routine. Its battery has an operational autonomy of at least 12 hours. Our goal was to make the device sensitive enough to detect even the slightest change of upper limb position. This report describes a monocentric clinical pilot study with the objective to demonstrate feasibility and reliability of physical data recorded with ActiMyo^®^ in a laboratory setting for non-ambulant DMD patients.

## Materials and Methods

The protocol for this trial and the TREND checklist are available as supporting information; see S1 TREND Checklist, S1 Protocol and S2 Protocol.

### Patients

The study was conducted between January 2012 and December 2012 at the Institute of Myology, at Hospital Pitié-Salpêtrière, in Paris (France) within the Pre-Acti protocol approved by the local Ethics Committee *(Comité de Protection des Personnes*, *Ile-de-France VI*, *80–11)* and by the French Regulatory Agency *(ANSM*, *B111169-10)*. Patients were recruited from the neuropaediatric and adult neuromuscular consultation at the Institute of Myology and by information spread through by the French Muscular Dystrophy Association (AFM). Before inclusion, all patients or their parental authorities provided signed informed consent.

Patients over 10 years of age with genetic confirmation of their neuromuscular disease were included. These patients had to be non-ambulant (*i*.*e*. unable to walk 10 meters without external aid) and had to be able to sit for at least 3 hours in the wheelchair.

The exclusion criteria were: cognitive impairment, occurrence of neurological, inflammatory, infectious, endocrine, or acute orthopedic disease in the previous month, scheduled surgery within 3 weeks of inclusion date, and occurrence of surgery of the upper limbs in the previous three months.

### Study design

Patients performed several tests while wearing the ActiMyo^®^ device in a laboratory setting at baseline and fifteen days later in order to analyze both feasibility and reliability. These tests involved the upper limb and consisted of tasks to quantify strength (using the MyoGrip and the MyoPinch [48–50]) and motor abilities (MoviPlate [42], Box and Block test [51], and a modified version of Minnesota dexterity test [52]), and tasks mimicking daily activities like typing and handwriting. All tests were done with both hands, starting with the dominant hand, three times (except for handwriting, which was done only once with the side normally used for this activity), and while wearing the ActiMyo^®^ on the wrist. The patients were video recorded throughout all the tasks.

### ActiMyo^®^

**Fig 1:**
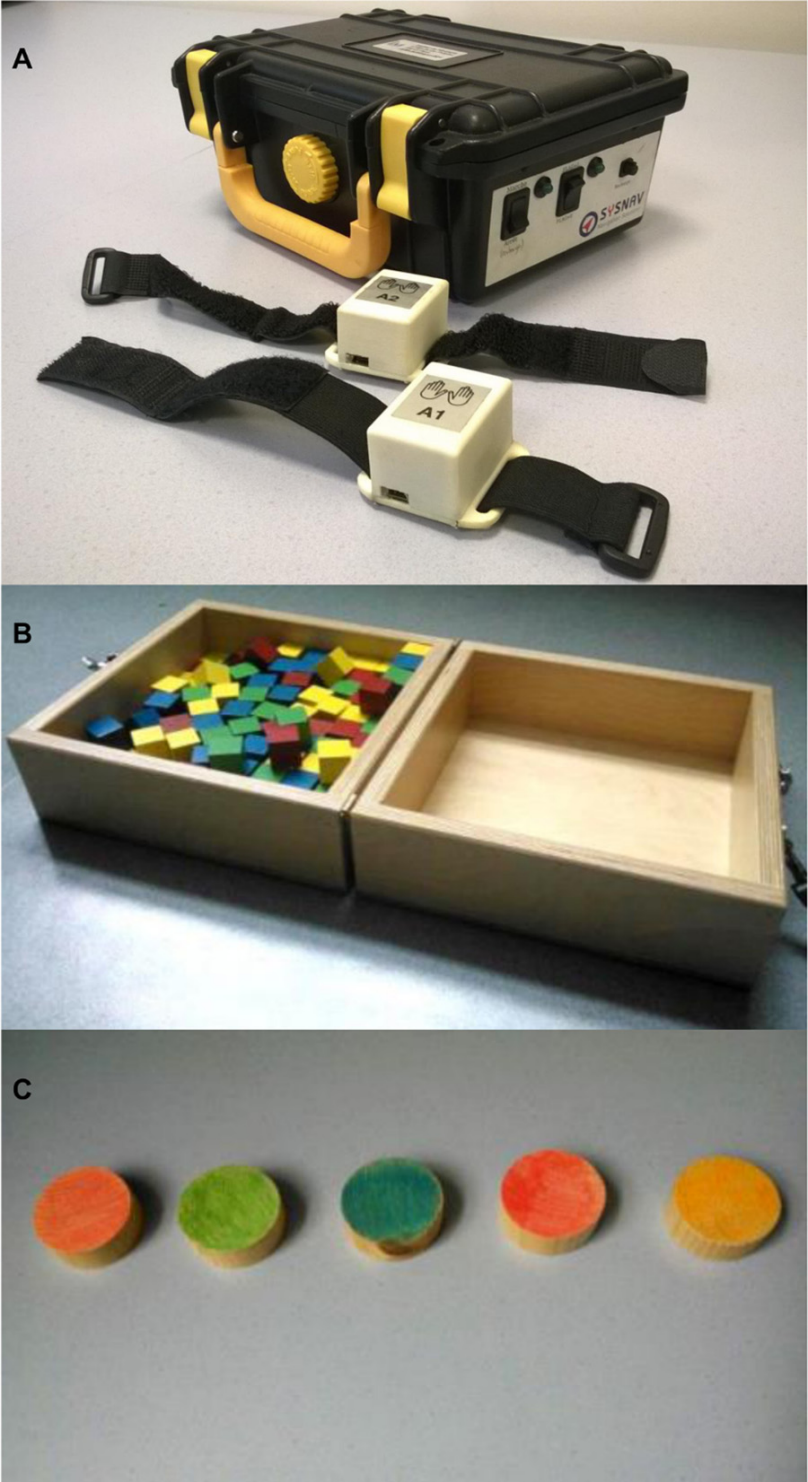
The tools. **(A)** First version of ActiMyo^®^, used in the current study. **(B)** Box and Block test. **(C)** Minnesota test with five discs.

A prototype version of the ActiMyo^®^ was used in this study. It consisted of two watch-like devices (length x width x height: 40 x 27 x 25 mm; weight: 38 grams) connected through Bluetooth to the recording station (210 x 145 x 90 mm) (Fig 1A). The watch-like devices contained a three-axis accelerometer, a three-axis gyroscope, and a three-axis magnetometer that recorded respectively the linear acceleration, the rotation rate (angular velocity), and the magnetic field in the three dimensions of space. The autonomy of the system was approximately 16 hours. Raw data were transmitted in real-time to the recording station nearby and were stored on a micro SD card.

During the tasks in the laboratory setting, the watch-like devices were placed on each wrist of the patient and the recording station was kept within the range of 4 meters, on a stable surface. The recording station was also equipped with a switch to record a binary signal to mark the beginning and the end of any specific event, as chosen by the evaluator.

The ActiMyo^®^ system records the following in the watch reference frame:

- The specific acceleration vector that indicates the instantaneous inertial acceleration for each axis (*Ax*, *Ay*, and *Az*). It is expressed in g-force (1 g = 9.81 m/sec^2^).
- The instantaneous angular velocity vector that indicates the instantaneous rotation of the wrist in the reference system of the watch (*Gx*, G*y*, and *Gz*). It is expressed in degrees per second (°/sec).
- The magnetic field vector that indicates the direction and strength of the local magnetic field (*Mx*, *My*, and *Mz*). It is expressed in gauss (in Paris, the Earth's magnetic field is approximately 0.45 gauss).

### Evaluations in laboratory setting

Patients were assessed using the MyoSet as previously reported [42,48,49]. Briefly, MyoPinch assessed keypinch strength, MyoGrip measured hand grip strength, and MoviPlate assessed the patients’ ability to move their hand using finger and wrist flexors and extensors to hit alternatively two targets of different heights placed in the sagittal plane for 30 seconds. For the Block and Box test (BBT) [51], the patient was asked to move 29-mm^3^ wooden cubes from one box to another one by one. In our study, this test was performed without the middle partition (Fig 1B) so that the test could be completed by most of the patients. Each trial lasted 60 seconds. The score was equal to the number of blocks moved. The Minnesota Test [52] is a test during which patient has to switch different wooden discs disposed on a table. We adapted this test taking into consideration the condition of the patients: we used only five discs in a row that must be turned over repeatedly during 60 seconds (Fig 1C). The score was equal to the number of turned discs. Daily life non-validated activities were chosen to reflect the relevant activities (typing, writing). The sentence to be hand written or typed on a PC keyboard was chosen to be simple and had no special characters (“*Les pompiers sont partis de la caserne avec leur camion rouge”*). The score for both tasks corresponded to the number of characters of the phrase written/typed one time per trial in 60 seconds.

### Data collection

Baseline medical information (clinical status, cardiac, respiratory, and orthopedic data) was collected at the first visit and any notable recent medical issue was documented at follow-up. Magneto-inertial data were analyzed using specific software developed by Sysnav Company using MatLab^®^ (R2009b, The MathWorks, Natick, MA).

### Signal processing and variable computation

The measurements were first calibrated (*i*.*e*. compensated to take into account the most relevant errors) before being processed to estimate other meaningful physical quantities. The main errors typically consisted of biases, and scale factor errors at the one-axis sensor level, and mis-orthogonality errors between axes and cross-coupling at the 3D sensor level. These errors were estimated by calibration procedure where the raw measurements were compared, either directly or indirectly (norm of vector, for instance) to a known and controlled quantity [53,54], either found in the nature (gravity, Earth magnetic field) or obtained through high-end motion stimulators. Once the data are calibrated, it is possible to deduce the orientation of the device in the Earth’s reference frame through attitude estimation algorithms [55,56]. Integrating the gyroscopes allow to estimate the short-term attitude variations. However, such a solution alone drifts over time. Accelerometers and magnetometers permitted to give an initialization point and to eliminate that drift as according to Wahba’s problem [57], the measurements of two independent vectors are sufficient to fully determine the orientation. Practically, an extended Kalman filter was chosen to implement the attitude estimation algorithm [58]. For the sake of interpretability, the estimated attitude was expressed as Euler angles. Given the orientation of the device, measured or estimated quantities could then be expressed either in the frame of the device, attached to the wrist, or in the Earth’s reference frame. The vertical and upward component of the acceleration in the Earth reference frame was interpreted as the anti-gravity component of the acceleration which was of particular clinical significance for weak neuromuscular patients. Many other parameters and variables (rotation rate around the forearm axis and the vertical axis, acceleration, torque…) were computed to reflect the movement of rotation of the forearm around the elbow based on the assumption that the elbow was fixed. For each of them, several statistical variables were tested (minimum, maximum, mean, median, standard deviation, norm). In order to select variables that seemed to be most suitable and consistent for the study of upper limbs, a preliminary analysis was done with the help of video recordings. Variables that seemed most reliable and clinically relevant were selected, taking into consideration the weakness and paucity of upper limb movements in DMD patients. The following variables were selected:

- The norm of the angular velocity (denoted ‖Ω‖) of the wrist wearing the device; it was expressed in degrees per second (°/sec) and directly obtained from the calibrated measurements. Its value does not depend on the frame of expression (sensor frame or Earth’s reference frame).
- The ratio of the vertical component of the acceleration to the overall acceleration (denoted vA); it was expressed without units. The ratio was taken once the acceleration was expressed in the Earth’s reference frame.
- A model-based computed power (denoted P) that corresponds to the mechanical power necessary to move the forearm based on the assumption that the elbow is not moving. It was calculated as the scalar product of the torque and the angular velocity, expressed in the Earth’s reference frame. An inertial matrix (expressed per unit of mass) was arbitrary chosen to model the forearm: the length of the forearm was set to 20 cm with a 3-cm radius. This matrix was the same for all patients regardless of their weight. This parameter was expressed in watts per unit of mass (W/kg), so that the unknown weight of the forearm did not interfere.
- The elevation rate (denoted dθ) corresponds to the temporal derivative of the elevation angle of the forearm, one of the three Euler angles used to represent the orientation of the device. This angle was taken between the horizontal plane and the direction of the forearm. The elevation rate thus represented the angular velocity at which the forearm lifted. It was expressed in degrees per second (°/sec).

### Statistical analysis

In order to assess a possible laterality effect, we compared the maximum value obtained for strength (grip and pinch) and function (MoviPlate score) between the dominant and non-dominant hand using the non-parametric Wilcoxon matched-pairs signed-rank test. The reliability of ActiMyo^®^ variables (‖Ω‖), vA, P, dθ) and task scores were assessed for each task first (average value over all repetitions of the task) and scores of all tasks were pooled to estimate the intra-class correlation coefficient (ICC). ICC was computed using a two-way random effect model (absolute agreement) for average measurements on all trials. Correlations between the functional scores (MoviPlate, BBT, Minnesota, PC typing, handwriting) and the ActiMyo^®^ variables were assessed using Spearman’s rank correlation coefficient as relationships between variables might not be linear. Data from all three trials per visit were used. All analyses were performed using the SPSS 19 statistical software (SPSS Inc., Chicago, IL). The limit of statistical significance was set to 0.05.

## Results

### Clinical features

**Fig 2:**
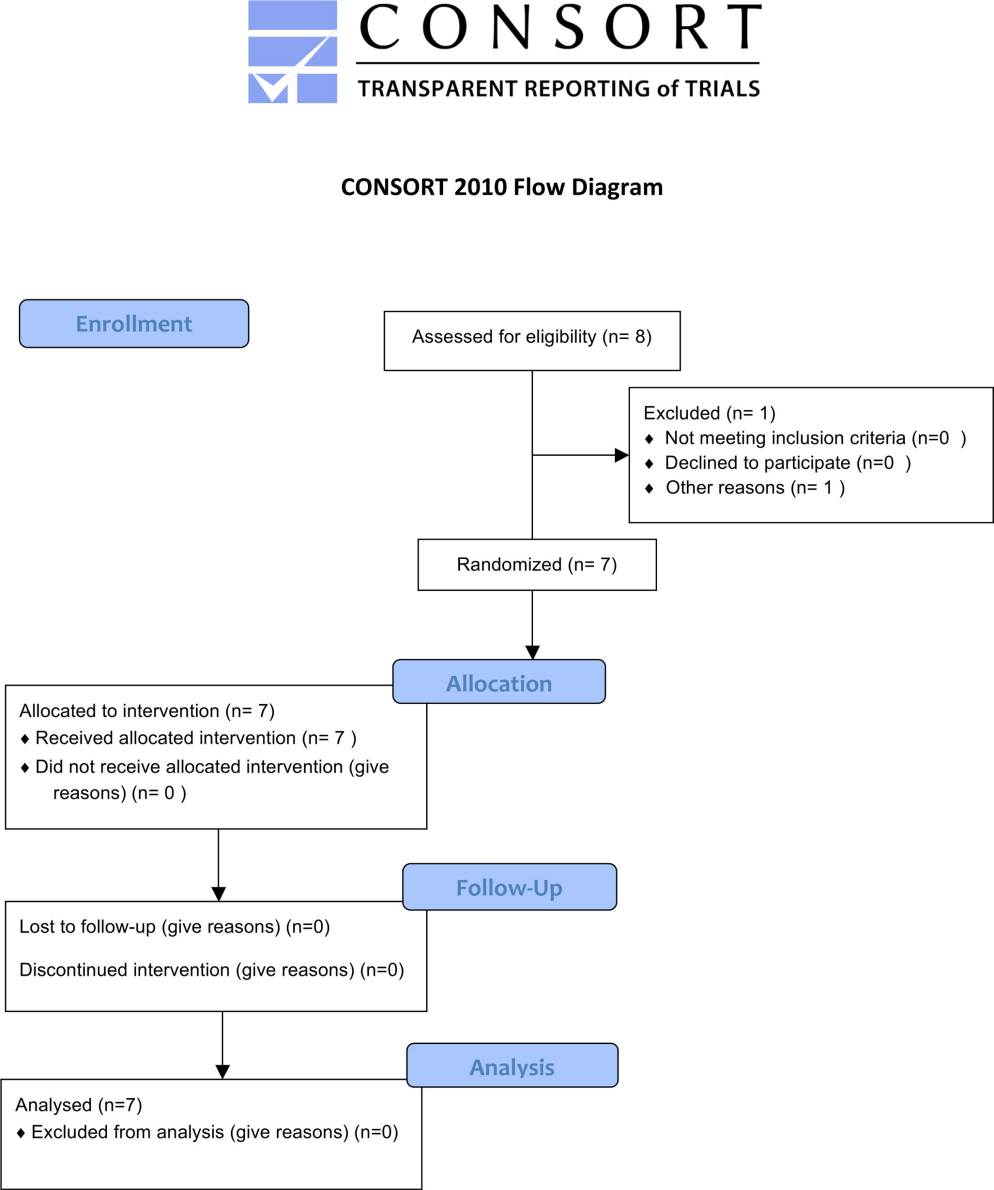
Patient flow-chart.

Seven DMD patients (genetically characterized: four with deletions, two with point mutations, and one with a duplication) were included in the study (Fig 2). The patients had a mean age of 18.5 ± 5.5 years. Clinical features are reported in Table 1.

**Table 1:** Clinical features of the DMD patients ordered by increasing age.

Patient	Age (years)	Mutation	Age at ambulation loss (years)	Brookescore	Non-invasive ventilation	Steroid treatment	Grip D (kg)	Pinch D (kg)
#1	10.5	del3-11	10.0	3	no	no	4.66	1.50
#2	15.5	c.10453_10454insC	12.0	2	no	yes	13.99	3.55
#6	16.0	del42-43	11.0	3	no	no	6.43	2.58
#4	18.0	c4084C>T	8.5	5	no	no	6.46	1.55
#5	20.0	dup48-49	9.0	6	no	no	1.13	1.22
#7	20.5	del49-50	8.0	6	nocturnal	no	0.62	0.18
#3	28.5	del51	11.0	6	continuous	no	0.72	0.30
Mean (SD)	18.5 (5.5)		10.0 (1.5)	4.4 (1.7)				

D—dominant side.

### Feasibility

Patients were able to complete most of the tasks (Table 2). Their primary limitation was due to their weakness and/or contractures at baseline. For the patient #6, the follow-up visit data was excluded due to technical problems.

**Table 2:** The maximal scores on tasks completed by each patient at the two visits sorted by the decreasing age.

Patients	MoviPlate D	MoviPlate ND	BBT D	BBT ND	Minnesota (n) D	Minnesota (n) ND	PC Typing (score/min)	Writing (score/min)
#1	54	51	52	50	69 (5)	74 (5)	61	87
#2	95	87	67	67	67 (5)	70 (5)	174	161
#6	67	55	36	32	61 (5)	16 (5)	99	87
#4	58	61	30	29	27 (5)	33 (5)	60	109
#5	31	43	NA	NA	38 (1)	43 (1)	NA	25
#7	35	38	NA	NA	NA	48 (1)	NA	NA
#3	29	25	NA	NA	11 (1)	20 (1)	NA	25

D—dominant hand, ND—non dominant hand, NA—not available, n—number of discs used.

The Minnesota test was adapted to the functional and muscular abilities of the patients. The three most affected patients (#3, #5, and #7) used only one disc because of their severe muscle weakness and upper limb contractures. Patients #1, #2, #4, and #6 performed the test with five discs. The analysis was done separately for the patients using five discs and those using only one. One patient could not perform the test with the dominant hand (even with the mentioned adaptations of the test).

The ActiMyo^®^ device was well tolerated by the patients, and it was never an obstacle in accomplishing the tasks demanded. ActiMyo^®^ data were recorded for all the tasks performed, even in the weakest patient.

### Dominance effect

The laterality effect was tested for all the patients in all the tasks, and no dominance effect was found (Table 3).

**Table 3:** Effect of dominance on functional task results.

	Dominant side			Non-dominant side			Side effect
	N	Mean	SD	N	Mean	SD	p-value
MyoGrip (kg)	7	4.86	4.79	7	4.20	3.82	0.176
MyoPinch (kg)	7	1.55	1.20	7	1.34	0.88	0.271
MoviPlate (score)	7	52.71	23.70	7	51.43	19.66	0.551
BBT (score)	4	46.25	16.66	4	44.50	17.64	0.109
Minnesota (score)	6	45.50	23.85	7	43.43	22.63	0.340
PC Typing (score/min)	4	98.55	53.64	NA	NA	NA	NA
Writing (score/min)	6	82.53	52.06	NA	NA	NA	NA

N—number of patients accomplishing the test; BBT—Box and Block test.

### Test–Retest reliability

**Fig 3:**
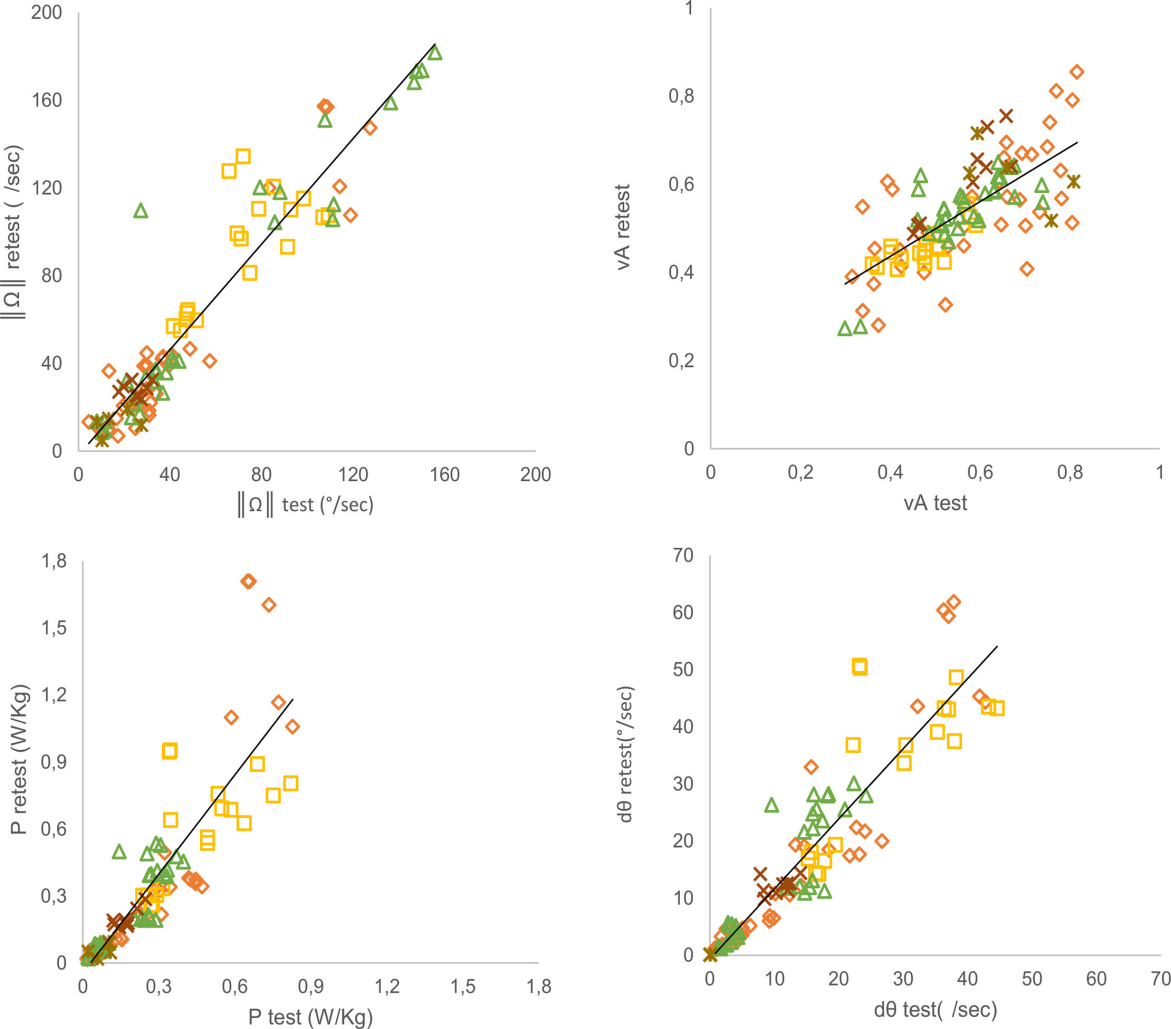
Reliability between test and retest for all the tasks evaluated for each ActiMyo^®^ variable. **◇** MoviPlate; **⬜** BBT; **△** Minnesota test; **✱** Writing;**×** PC typing.

All ActiMyo^®^ variables showed high to very high reliability as assessed using ICC values (all ICC ≥ 0.8; Table 4). The correlations between test and retest for each variable are displayed in Fig 3. Analyzed by tasks, all variables showed high to very high reliability for MoviPlate and for Minnesota. Moderate to high reliability was observed for the BBT, PC typing, and writing.

**Table 4:** Reliability of tasks between test and retest of ActiMyo^®^ inertial variables and task scores.

	All tasks together		Moviplate		BBT		Minnesota		PC typing		Writing	
	N	ICC	N	ICC	N	ICC	N	ICC	N	ICC	N	ICC
‖Ω‖	99	0.950	36	0.958	18	0.700	31	0.958	9	0.147	5	0.482
P	99	0.841	36	0.816	18	0.679	31	0.860	9	0.829	5	0.648
vA	99	0.818	36	0.794	18	0.786	31	0.857	9	0.889	5	NM
dθ	99	0.925	36	0.933	18	0.783	31	0.884	9	0.446	5	0.607
Scores	-	-	36	0.984	18	0.970	31	0.970	9	0.954	5	0.987

BBT—Box and Block test; N—number of paired trials between visits for both dominant and non-dominant hands of all the patients; NM—not measurable.

### Correlation between the ActiMyo^®^ variables and the functional scores (Table 5)

**Fig 4:**
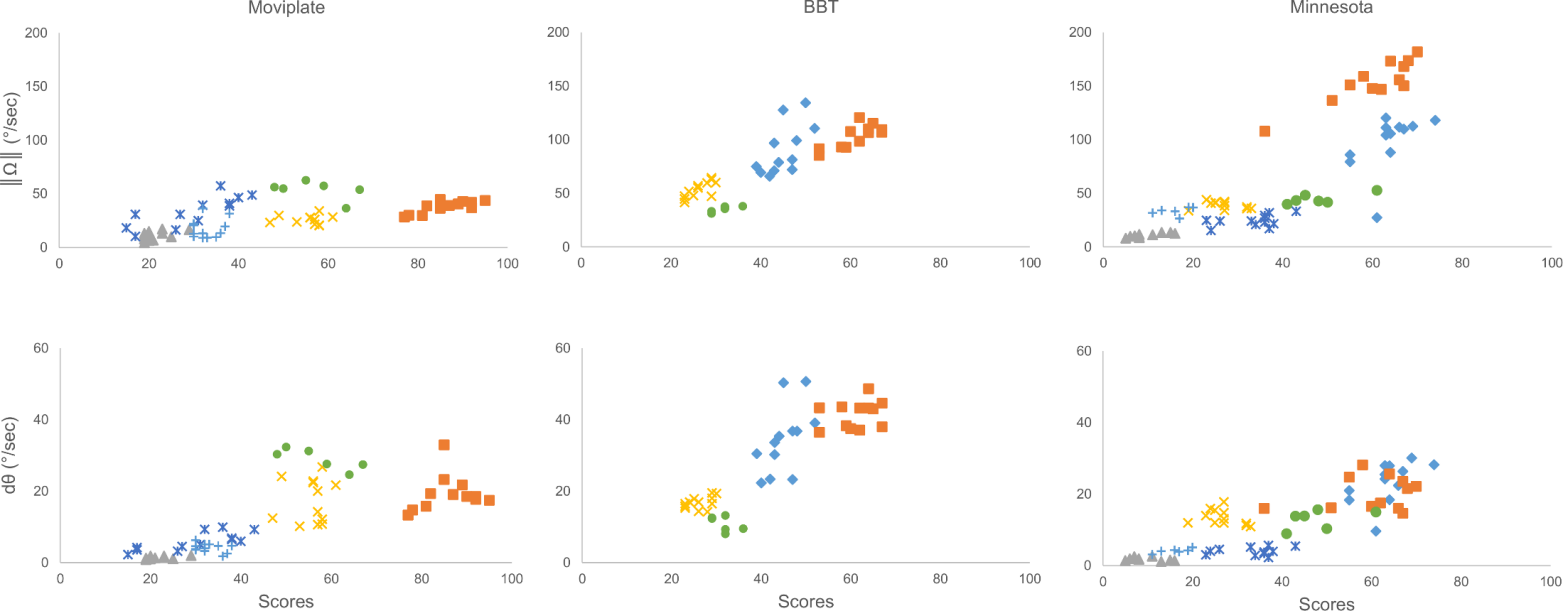
Correlation between the ActiMyo^®^ variables and the functional tests’ scores. **◆** Patient 1; ■ Patient 2; ▲ Patient 3; × Patient 4; * Patient 5; ● Patient 6; + Patient 7. Correlations between ‖Ω‖ and scores for the tasks **(A)** Moviplate, **(B)** Box and Block test, and **(C)** Minnesota. Correlation between dθ and scores for the tasks **(D)** Moviplate, **(E)** Box and Block test, and **(F)** Minnesota.

For the MoviPlate and BBT, all variables were significantly correlated with the functional scores. ‖Ω‖, P, and dθ were significantly correlated with the Minnesota scores and with the writing task (Fig 4). MoviPlate data for patient #1 had to be discarded due to a technical issue.

**Table 5:** Correlation between the ActiMyo^®^ variables and the functional scores.

	MoviPlate^§^		BBT			Minnesota Patients: 1-2-4-6		Minnesota Patients:3-5-7		PC Typing		Writing	
	N	ρ	N	ρ		N	ρ	N	ρ	N	ρ	N	ρ
‖Ω‖	66	0.671*	42		0.830*	42	0.765*	28	0.573*	21	-0.201	11	0.665*
P	66	0.858*	42		0.820*	42	0.719*	28	0.579*	21	0.157	11	0.656*
vA	66	0.677*	42		-0.843*	42	-0.082	28	0.076	21	-0.678*	11	-0.114
dθ	66	0.850*	42		0.823*	42	0.679*	28	0.616*	21	0.298*	11	0.715

BBT—Box and Block test; N—number of trials for both dominant and non-dominant hands of all the patients at both visits.

§ Without patient 1

* Significance level p < 0.05.

## Discussion

In this study we compared use of a magneto-inertial-based movement monitor, ActiMyo^®^, with previously described functional assessments in boys and adults with Duchenne muscular dystrophy to quantitatively detect and measure a various range of upper limbs movements observed in non-ambulant patients.

One of the limitations of this study was the small number of patients evaluated. This is mainly due to the fact that only one prototype was available at the time the study was initiated and that all analyses were manually performed. However many data are coming about of large cohort of patients in longitudinal international clinical trials. The data is coming soon to assess muscle function of these patients in their daily lives. The group evaluated was chosen heterogeneous. Severity of symptoms ranged from the patient who had recently lost ambulation (patient #1) to the older patient who required continuous ventilation (patient #3). Due to the general muscle weakness of these patients, tools were used to reliably assess low levels of distal strength and function. It is known that hand grip and pinch strength correlate with the global hand strength, which is inversely correlated with functional capacity [59]. All patients from our study performed previously validated strength tests (MyoPinch and MyoGrip), and the results from these tests reflected the clinical status of the patients defined by the functional Brooke score [60].

We did not find any side effect between the dominant and non-dominant hands in our population. This is in line with our previous findings [42]. Other authors demonstrated that differences between dominant and non-dominant sides may partially be counteracted by physical activity [59]. In adult Duchenne patients, Bartels *et al*. reported a significant but relatively small difference in MFM D3 upper limb score between sides [61]. In our study, patients generally performed better with their dominant side but this was not statistically significant. This lack of statistically significant difference was likely due to the small size of the population and to the fact that the majority of the patients presented with an advanced stage of the disease. ActiMyo^®^ data from both hands were pooled for the analyses.

Reliability was difficult to establish when quantifying the different parameters of movement as measured by ActiMyo^®^, since patients may change motor strategy from one trial to another. The reliability of ‖Ω‖ was comparable to each of the scores of the Minnesota test and to Moviplate. In other tasks, additional non-efficient movements of patients such as trying to catch a block in the BBT or hesitating during PC typing had a big impact on the reliability in this limited set of experiences.

The ratio of the vertical component of the acceleration on the whole acceleration norm (*vA*) and the elevation rate (dθ) highlight matching lifting and lowering movements of the wrist and forearm in the vertical direction. The mean elevation rate (dθ) covers a clinically significant outcome, namely the ability of the patient to lift the arm in the vertical axis, which is crucial for self-feeding, drinking, teeth brushing, scratching, and other tasks. Antigravity movements are a key component of several validated scales and scoring, such as Manual Muscle Testing [62], Brooke score [60,63], Motor Function Measure [41], Hammersmith scale [64], Performance of Upper Limb [43], CHOP INTEND [65], ABILHAND [66] and quality of life INQol questionnaires [67]. dθ reflected the lifting movement involved in rotating the arm, whereas *vA* reflected translational movements in the vertical direction. Since most weak patients use their elbows as lever arms, dθ probably better reflects the dynamics of movement than *vA*.

The rotation rate (‖Ω‖) and the model-based power *P* were directly linked to rotations of the wrist and forearm, respectively (BBT, writing test, and the Minnesota test). *P* was the scalar product of the motor torque and angular velocity. The torque was anti-gravitational in our case and was applied to lift the forearm. The torque was higher when the lifting movement was near the horizontal plane and may be particularly relevant for weak patients.

In summary, the ActiMyo^®^ variables were well representative of movements performed during the tasks and well correlated well with the scores obtained using other previously validated tests, which makes these variables potential outcome measures in neuromuscular patients. The norm of the angular velocity (‖Ω‖) and mean of the elevation rate (dθ) are the most promising variables since they presented good reliability and very good correlation with the scores obtained on other tasks, with the exception of PC typing. Detection of the mean elevation rate using the magneto-inertial device allows a precise and objective quantification of muscular activity during their daily life. The variable *P* is expected to be sensitive to small movements as it is the product of the angular velocity. The validity of these variables are now being assessed in a home environment. Various factors other than motor weakness, like the parental stimulation or socioeconomic environment, could interfere with a patient’s activity [68,69]. However, we expect that for non-ambulant DMD patients, weakness is the main determinant of upper limb activity and that long term recording possible with ActiMyo^®^ will help to low-pass filter daily variability and thus will provide a robust estimation of the trend of functional changes.

Further validation is required to determine the feasibility of the use of ActiMyo^®^ in a home environment. Possible confounding issues will be the feasibility of automatically detecting wheelchair and caregiver-aided movement. This work is ongoing in several patients with various neuromuscular disorders. Additional steps necessary for outcome measure qualification include the demonstration of reliability on day-to-day and week-to-week bases, the evaluation of the sensitivity to change and the correlation with clinically meaningful milestones in a multicentric setting. ActiMyo^®^ is now and integral part of several European and American natural history studies (ClinicalTrials.gov Identifier NCT01385917 for DMD and NCT 02057705D for Myotubular Myopathy) and is also being used in a therapeutic trial in DMD (ClinicalTrials.gov Identifier NCT01826474).

### Perspectives

The Pre-Acti protocol was a feasibility study of ActiMyo^®^ in recording movements of non-ambulant patients. It was critical that the device detected very small movements because the weakest movements allow a certain degree of autonomy for patients with neuromuscular diseases such as DMD. Much work was invested into improving the system as software and hardware changes were made during the study. ActiMyo^®^ has evolved into a redesigned and upgraded final version (S1 Fig) that is shipped in a watertight durable case. It now consists of two watch-like devices and a docking station with data stored in an internal memory inside each watch-like device and transferred in the evening to the docking station through electrical contacts. Data collected in the docking station are then stored on a USB drive that can hold up to three months of data. Charging of the sensors battery is performed when the watch-like devices are put on the docking station each night. The main advantage of the newer version is the reduction of the size and the weight of the device. This size reduction was possible because less battery life is required to operate them without the Bluetooth connection, which was the most power consuming component of the prototype version. The downside of ActiMyo^®^ could be its production price which is partly due to the high quality sensors it contains and the semi-automatic data analysis that is currently performed. Nevertheless, the first devices manufactured in small series will allow us to gather longitudinal home data in non-ambulant patients with neuromuscular diseases in order to assess efficiency and consistency of this magneto-inertial-based movement monitor in long-term recordings. New technical requirements will also be considered in the development of ActiMyo^®^ for other applications such as use by ambulant patients. Further use of ActiMyo^®^ will lead to a qualitative and quantitative assessment of usability of this innovative device in various populations.

## Supporting Information

S1 TREND Checklist (PDF)

S1 Fig Principal components of the final version of ActiMyo^®^. (TIF)

S1 Protocol Trial Protocol (French). (PDF)

S2 Protocol Trial Protocol (English). (PDF)
