# Supplementary figures and images for: A Movement Monitor Based on Magneto-Inertial Sensors for Non-Ambulant Patients with Duchenne Muscular Dystrophy: A Pilot Study in Controlled Environment
Source: PLoS One. 2016 Jun 7;11(6):e0156696. doi: 10.1371/journal.pone.0156696 (PMC4896626; doi:10.1371/journal.pone.0156696)

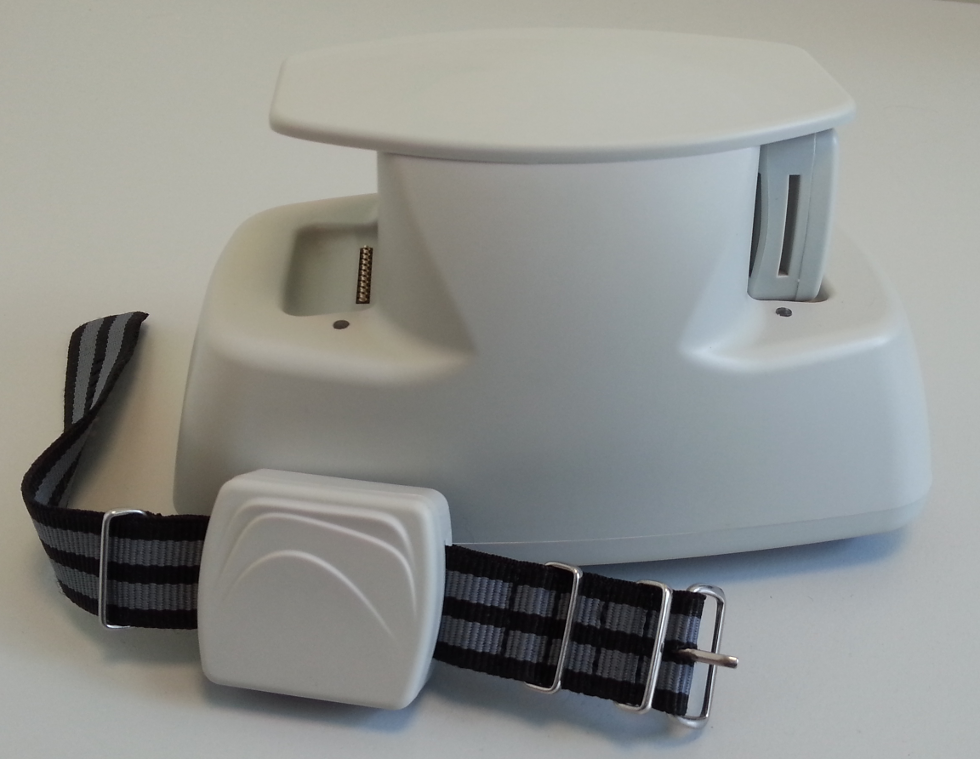

Supplement: S1 Fig — (TIF) [file pone.0156696.s002.tif]
