# Supplementary material for: A Movement Monitor Based on Magneto-Inertial Sensors for Non-Ambulant Patients with Duchenne Muscular Dystrophy: A Pilot Study in Controlled Environment
Source: PLoS One. 2016 Jun 7;11(6):e0156696. doi: 10.1371/journal.pone.0156696 (PMC4896626; doi:10.1371/journal.pone.0156696)

Projet de recherche biomédicale

Mesure continue de l'activité pour l'évaluation clinique à domicile des patients non ambulants atteints d'une pathologie neuromusculaire

Etude Pré-Acti

Promoteur : Institut de Myologie – GH Pitié-Salpêtrière – 75651 Paris Cedex 13

Tel: 01-42-16-66-46 Fax: 01-42-16-58-87

Investigateur principal : Dr. Laurent Servais - Institut de Myologie

Tel: 01-42-16-56-70 Fax: 01-42-16-58-56

Co-investigateurs : Anne-Gaelle LE MOING

Scientifiques associés : Jean Yves HOGREL, Andrea TOTOESCU, Mohamed BENALI, Amélie MORAUX, Aurélie CANAL.

Plan expérimental : Evaluation et validation de système de mesure

Type d'étude : multicentrique

Lieux de l'étude :

- Institut de Myologie
- CHU Amiens

N° d'enregistrement AFSSAPS : 2011-A00795-36

SOMMAIRE

1. RESUMÉ 4
2. INTRODUCTION 5
3. CONTEXTE ET JUSTIFICATION DE LA DEMANDE 8
4. OBJECTFS 9
5. METHODOLOGIE 9
6. CRITERES D'INCLUSION 9
7. CRITERES D'EXCLUSION 10
8. MODALITÉS PRATIQUES 11
8.1. Déroulement général de l'étude : 12
8.2. Déroulement des évaluations 12
9. DESCRIPTION DES EVALUATIONS 13
10. RECUEIL DE DONNEES 16
11. BENEFICE ATTENDU 16
12. DUREE D'EXCLUSION 16
13. GESTION DES SORTIES D'ESSAI 17
14. GESTION DES EVENEMENTS INDESIRABLES 17
15. INDEMNISATION 17
16. ENGAGEMENT DE L'INVESTIGATEUR 18
17. LETTRE D'INFORMATION ET CONSENTEMENT ECLAIRE 18
18. LA GESTION DES DONNES 19
18.1. La confidentialité 19
18.2. L'analyse des donnés 20
18.3. L'archivage des documents 20
19. AMENDEMENTS AU PROTOCOL 21

20. DUREE DE L'ETUDE 21

21. RAPPORT FINAL ET PUBLICATIONS 21

22. ANNEXES 22

22.1. ANNEXE 1 22

22.2. ANNEXE 2 22

22.3. ANNEXE 3 24

22.4. ANNEXE 4 24

22.5. ANNEXE 5 25

22.6. ANNEXE 6 25

22.7. ANNEXE 7 26

1. RESUMÉ

Le besoin de mesures spécifiquement adaptées pour évaluer la force et la fonction résiduelle aux membres supérieurs de patients non ambulants atteints des maladies neuromusculaires (surtout la Dystrophie musculaire de Duchenne et l'Amyotrophie spinale) est toujours d'actualité.

À la fois dans une visée de surveillance régulière de l'évolution clinique des patients non ambulants, atteints de maladies neuromusculaires, mais aussi pour permettre de doter la recherche d'un système de mesure quantitative puissant, le projet **Pré-Acti** propose l'évaluation d'un système complet de mesure de l'activité musculaire en continu et à domicile de patients non ambulants.

Le principe des mesures réside dans l'utilisation de l'accélérométrie et des capteurs magnéto-inertiels, une technologie innovante d'estimation de la vitesse linéaire et angulaire inventée et développée par la société SYSNAV.

Le système a pour but de mesurer l'activité physique d'un patient à partir de l'extraction, dans les trois axes de l'espace, des données issues de capteurs fixés sur l'un de ses membres supérieurs.

Dix patients non ambulants seront inclus et évalués à l'inclusion et 14 jours plus tard. Les patients porteront un petit dispositif plastique sous forme de montre pendant les évaluations et aussi à domicile pendant 14 jours.

Par le présent protocole, nous voulons démontrer que l'enregistrement simultané des accélérations linéaires et des vitesses angulaires dans les trois axes chez les patients myopathes non ambulants à domicile permet d'estimer de manière simple, fiable, reproductible et précise l'activité aux membres supérieurs. Dès lors, le but d'**ActiMyo** est de proposer une mesure d'*outcome* primaire susceptible de servir dans les essais thérapeutiques et dans le suivi clinique des patients.

2. INTRODUCTION

La Dystrophie musculaire de Duchenne (DMD) est à ce jour la plus fréquente dystrophie musculaire chez l'enfant. C'est une maladie génétique liée à l'X dont l'incidence est de 1/3500 naissances males (Emery 1991)[1] avec une évolution progressive, conduisant à la perte de la marche vers la deuxième décennie et le décès vers la quatrième décennie (Kohler et al. 2009)[2]. Elle touche l'ensemble des muscles de l'organisme avec une limitation progressive de l'utilisation des membres supérieurs. Le traitement par glucocorticoïdes est le seul dont l'effet est à ce jour démontré de ralentir la régression de la force et la puissance musculaire (Manzur et al., 2008)[3].

L'absence de la dystrophine dans la DMD est due à des mutations variées du gène de la dystrophine, interrompant le cadre de lecture. Compte tenu de l'organisation en 79 exons du gène, le cadre de lecture peut être rétabli par l'exclusion d'un ou plusieurs exons si la phase locale s'y prête. C'est ce qu'on appelle le saut d'exon thérapeutique (*exon skipping*) (Aartsma-Rus et al., 2002)[4]. A ce jour, plusieurs essais de thérapie par saut d'exons sont déjà en cours ou vont bientôt démarrer dans des essais cliniques (Nelson et al., 2009)[5]. Un aspect très important est la nécessité de baser la construction des essais thérapeutiques sur des mesures fiables et validées, c'est-à-dire reproductibles, sensibles, objectives.

A l'issue du workshop treat-NMD du 11 juillet 2007, le besoin de mesures spécifiquement adaptées pour évaluer la force résiduelle aux membres supérieurs de patients non ambulants a

---

[1] Emery, A. E. (1991). "Population frequencies of inherited neuromuscular diseases--a world survey." Neuromuscul Disord **1**(1): 19-29.

[2] Kohler, M., C. F. Clarenbach, et al. (2009). "Disability and survival in Duchenne muscular dystrophy." J Neurol Neurosurg Psychiatry **80**(3): 320-325.

[3] Manzur AY, Kuntzer T, Pike M, Swan A. Glucocorticoid corticosteroids for Duchenne muscular dystrophy. *Cochrane Database Syst Rev* 2008; **1:** CD003725.

[4] Annemieke Aartsma-Rus, Mattie Bremmer-Bout, Anneke A. M. Janson, Johan T. den Dunnen, Gert-Jan B. van Ommen, Judith C. T. van Deutekom, Targeted exon skipping as a potential gene correction therapy for Duchenne muscular dystrophy, Neuromuscular Disorders, Volume 12, Supplement 1, October 2002, Pages S71-S77, ISSN 0960-8966, DOI: 10.1016/S0960-8966(02)00086-X.

[5] Nelson SF, Crosbie RH, Miceli MC, Spencer MJ. , Emerging genetic therapies to treat Duchenne muscular dystrophy., Curr Opin Neurol. 2009 Oct;22(5):532-8.

été soulevé (Mercuri et al, 2008)[6]. Par conséquent, différentes échelles ont été proposées, parmi lesquelles le *Hammersmith Functional Motor Scale* (HFMS) (Scott et al., 1982)[7], l'*Egen Klassification Scale* (EK) (Steffensen et al., 2001)[8], et le *Motor Function Measure* (MFM) (Berard et al.,2006)[9]. L'avantage des ces différentes échelles est qu'elles évaluent de manière complète la fonction des membres supérieurs et ce, avec un minimum de matériel. Néanmoins, un réel inconvénient est le temps nécessaire à leur réalisation, la dépendance à la motivation du sujet et aussi la dimension subjective de la mesure. Un autre outil est en cours de validation à l'Institut de Myologie pour l'évaluation clinique du membre supérieur des patients DMD (Servais et al., 2010)[10].

La mesure manuelle de la force a récemment été proposée comme paramètre d'évolution de la maladie (Mattar et al., 2008)[11].

Par ailleurs, un problème similaire se pose pour d'autres maladies neuromusculaires privant parfois les sujets non ambulants de participer à des essais thérapeutiques. C'est le cas par exemple du saut d'exon 51 pour lequel l'essai de pharmacocinétique va seulement débuter chez les patients non ambulants, alors que ces essais sont en phase 3 chez les ambulants.

---

[6] Mercuri, E., Mayhew, A., Muntoni, F., Messina, S., Straub, V., Van Ommen, G.J., Voit, T., Bertini, E., Bushby, K. (2008). Towards harmonisation of outcome measures for DMD and SMA within TREAT-NMD; report of three expert workshops: TREAT-NMD/ENMC workshop on outcome measures, 12th--13th May 2007, Naarden, The Netherlands; TREAT-NMD workshop on outcome measures in experimental trials for DMD, 30th June--1st July 2007, Naarden, The Netherlands; conjoint Institute of Myology TREAT-NMD meeting on physical activity monitoring in neuromuscular disorders, 11th July 2007, Paris, France. *Neuromuscul. Disord. 18*, 894-903.

[7] Scott OM, Hyde SA, Goddard C, Dubowitz V. Quantitation of muscle function in children: a prospective study in Duchenne muscular dystrophy. Muscle Nerve 1982;5:291–301.

[8] Steffensen B, Hyde S, Lyager S, Mattsson E. Validity of the EK scale: a functional assessment of non-ambulatory individuals with Duchenne muscular dystrophy or spinal muscular atrophy. Physiother Res Int. 2001;6:119-134.

[9] Bérard, C., Payan, C., Fermanian, J., Girardot, F. (2006). A motor function measurement scale for neuromuscular diseases - description and validation study. *Rev. Neurol. (Paris) 162*, 485-493.

[10] L. Servais, A. Canal, N. De Coninck, A. Mesdelices, I. Desguerres, S. Quijnao-Roy, S. Mahmoudi, B. Estournet, M. Mayer, C. Themar Noel, T. Voit, J.Y. Hogrel, Upper limb evaluation in non-ambulatory patients with neuromusculardisorders, Neuromuscular Disorders 20 (2010) 596–68

[11] Mattar, F.L., Sobreira, C. (2008). Hand weakness in Duchenne muscular dystrophy and its relation to physical disability. *Neuromuscul. Disord. 18*, 193-198.

La réalisation des essais thérapeutiques pour les patients non ambulants est plutôt limitée par le manque des outils de mesures d'*outcome* pour ces patients.

A ce jour, il n'y a donc pas de mesure clinique simple adaptée aux patients atteints de maladies neuromusculaires non ambulants.

L'actimétrie consiste à mesurer l'activité d'un sujet pendant une période de temps donnée. Elle peut se réaliser au moyen de différentes approches (combinées ou non), parmi lesquelles l'accélérométrie. L'accélérométrie consiste à enregistrer les accélérations subies par l'accéléromètre, idéalement dans les trois axes de l'espace. Fixé au membre supérieur chez un patient non ambulant, l'accéléromètre permet d'enregistrer les accélérations de ce dernier, et donc de déduire les forces développées par le sujet pour mobiliser son membre supérieur, à condition bien sûr de connaitre son orientation, ce qui peut être réalisé grâce à des gyroscopes. Cette approche non invasive dans le suivi des patients est particulièrement intéressante puisqu'elle est réalisée chez des sujets dans leur environnement soit le domicile ou le lieu de vie habituel. La mesure a lieu lors des activités quotidiennes, elle est donc un reflet plus direct de la « vraie vie » du sujet. Par ailleurs, elle ne requière ni la motivation, ni la collaboration active du sujet, ce qui, chez les patients atteints de DMD présentant un retard intellectuel et/ou des troubles de l'attention, est un atout certain.

Pré-Acti est un projet développé par l'Institut de Myologie en collaboration avec la société SYSNAV.

Dans le cadre des maladies neuromusculaires, l'actimétrie par accélérométrie n'est à l'essai que pour les patients ambulants, dans le but de quantifier les épisodes de marche. (Jeannet et al, 2011)[12].

L'objectif de cette étude est donc de développer un outil capable de démontrer l'impact clinique d'une intervention dans la vie quotidienne du patient, composante essentielle de la

---

[12] Pierre-Yves Jeannet, Kamiar Aminian, Clemens Bloetzer, Bijan Najafi, Anisoara Paraschiv-Ionescu, Continuous monitoring and quantification of multiple parameters of daily physical activity in ambulatory Duchenne muscular dystrophy patients, European Journal of Paediatric Neurology, Volume 15, Issue 1, January 2011, Pages 40-47

médecine et de la réadaptation (Bonato, 2005)[13], et en même temps, d'améliorer la surveillance de l'évolution de la maladie.

3. CONTEXTE ET JUSTIFICATION DE LA DEMANDE

Comme nous l'avons mentionné, il est indispensable de définir une mesure clinique simple, fiable et reproductible de la fonction musculaire pour évaluer l'effet de nouvelles approches thérapeutiques chez des patients ayant une atteinte neuromusculaire (surtout DMD, SMA…).

L'accéléromètrie a été déjà mise en application pour l'évaluation de l'activité physique chez les enfants obèses (Tanaka et al., 2007)[14], les personnes âgées (Auvinet et al., 2003)[15] et aussi appliquée pour l'évaluation et la surveillance des personnes atteintes de la maladie de Parkinson (Giuffrida et al., 2009)[16], (Patel et al., 2007)[17]. L'évolution est déjà sensible pour la prise en compte des myopathies, et les mesures d'actimétrie ont fait récemment des progrès conséquents (Servais et al., 2009)[18].

---

[13] Paolo Bonato, Advances in wearable technology and applications in physical medicine and rehabilitation, Journal of NeuroEngineering and Rehabilitation 2 (2005) 1–4.

[14] Chiaki Tanaka, Shigeho Tanaka, Junko Kawahara, and Taishi Midorikawa, *Triaxial accelerometry for assessment of physical activity in young children.* Obesity, 2007. **15**(5): p. 1233-1241.

[15] Bernard Auvinet, Gilles Berrut, Claude Touzard, Laurent Moutel, Nadine Collet, Denis Chaleil, Eric Barrey, *Gait abnormalities in elderly fallers.* Journal of Aging and Physical Activity, 2003. **11**(1): p. 40-52.

[16] Giuffrida, J.P., et al., *Clinically Deployable Kinesia (TM) Technology for Automated Tremor Assessment.* Movement Disorders, 2009. **24**(5): p. 723-730.

[17] Shyamal Patel, Konrad Lorincz, Richard Hughes, Nancy Huggins, John H. Growdon, Matt Welsh, Paolo Bonato, *Analysis of feature space for monitoring persons with Parkinson's disease with application to a wireless wearable sensor system.* 2007 Annual International Conference of the Ieee Engineering in Medicine and Biology Society, Vols 1-16, 2007: p. 6291-6294.

[18] Servais L, Canal A, Hogrel JY, Voit T, *Quantification of upper limb activity in non ambulant boys with Duchenne muscular dystrophy using accelerometry: A proof-of-concept study.* Neuromuscular Disorders, 2009. **19**(8-9): p. 602-603.

4. OBJECTFS

Pré-Acti est une étude de validation. Elle consiste à mettre en évidence la pertinence d'une mesure clinique à domicile utilisant ActiMyo chez les patients atteints de maladie neuromusculaires non ambulants.

Ce protocole a pour but la démonstration de :

1) la faisabilité

2) la reproductibilité

3) la validation des données enregistrées par ActiMyo.

Cette étude a donc pour objectif d'envisager une nouvelle mesure clinique adaptée aux patients atteints de maladies neuromusculaires non ambulants.

5. METHODOLOGIE

Nous prévoyons d'inclure 10 patients non ambulants atteints des maladies neuromusculaires (DMD, SMA,…) âgés de plus de 10 ans. Les patients non-ambulants suivis à l'Institut de Myologie et dans le service de neuropediatrie au CHU d'Amiens seront sollicités pour participer à cette étude. Ils seront sélectionnés par le docteur Laurent SERVAIS à l'Institut de Myologie et par le docteur Anne-Gaelle Le MOING à l'Hôpital d'Amiens.

Les évaluations musculaires seront réalisées dans le laboratoire de physiologie et d'évaluation neuromusculaire, dirigé par Jean-Yves HOGREL, à l'Institut de Myologie.

L'ensemble des tests sera réalisé pour les patients neuromusculaires lors d'une visite programmée à l'Institut de Myologie.

Les patients désirant participer à l'étude recevront une copie de la note d'information, une lecture leur sera faite au cours d'une rencontre avec l'un des investigateurs. Une fois cette lecture faite et le consentement signé, les patients pourront être inclus dans l'étude.

6. CRITERES D'INCLUSION

Les patients doivent remplir les critères suivants :

- âgé de plus de 10 ans
- diagnostique d'une maladie neuromusculaire (DMD, SMA…) confirmée par la génétique ou par une biopsie musculaire.
- incapables de marcher 10 mètres sans aide humaine extérieure, ou canne.

- capables de tenir assis au fauteuil roulant pendant au moins trois heures.
- affilié à un régime de sécurité sociale
- consentement éclairé signé

7. CRITERES D'EXCLUSION

- Troubles cognitifs trop importants, limitant la compréhension des taches à réaliser
- Pathologie neurologique, inflammatoire, infectieuse, endocrinienne, ou orthopédique aigue dans le mois précédent l'inclusion.
- Chirurgie programmée dans les 3 semaines suivant l'inclusion
- Chirurgie au niveau de membres supérieurs dans les 3 mois précédent l'inclusion
- Femmes enceintes ou allaitantes

8. MODALITÉS PRATIQUES

Tableau 1. Calendrier des événements

		J0	J0 – J14	J14
Environnement		contrôlé	libre	contrôlé
**Recueil des données médicales**		x		
**Evénements intercurrents**				x
**Testes de force**	*Hand Grip* test	x		x
	Pinch Test	x		x
**Taches standardisées (scores)**<br><br>+ ActiMyo	*Box and block* test	x		x
	Minnesota test (variante)	x		x
	Moviplate	x		x
**Testes de la vie quotidienne (scores)** + ActiMyo	Taper à l'ordinateur une phrase	x		x
**Mouvements libres**<br><br>+ ActiMyo	Boutonner une veste	x		x
	Prendre le repas de midi	x		x
	Se brosser les dents	x		x
	Ecrire une phrase	x		x
ActiMyo en continu (environ 15h/j)<br><br>+ Journal			x	

8.1. Déroulement général de l’étude :

Durant leur première visite, les patients rencontreront un médecin investigateur, qui leur donnera la note d’information à lire et répondra à leurs questions. Le consentement éclairé sera ensuite recueilli et les données médicales seront notées (poids, taille, pathologie, score de Brooke et Walton, antécédents médicochirugicaux). Les patients recevront toutes les informations nécessaires concernant l’utilisation du dispositif **Actimyo** (voir plus bas). Il sera ensuite demandé aux patients de réaliser un ensemble de tests aux membres supérieurs, tout en enregistrant les accélérations et les vitesses angulaires par le dispositif **Actimyo**. L’ensemble des tests dure environ 3 heures. Cette première phase a pour but de corréler les mesures réalisées avec les résultats dans des taches validées en environnement contrôlé.

Par la suite, les patients seront formés afin de pouvoir porter **Actimyo** pendant deux semaines consécutives. Les enregistrements ne se feront que la journée, les batteries de l’appareil devant être rechargées la nuit. Il sera demandé au patient d’ôter l’appareil pour se baigner pour éviter toute immersion. Au total, environ 15 heures d’activité habituelle seront enregistrées par jour dans l’environnement du sujet. Durant toute cette période, le patient tiendra un journal de ses activités quotidiennes, en notant lui-même ou via un proche, l’heure de début et de fin de l’activité (par exemple se brosser les dents, manger, utiliser l’ordinateur, s’habiller). [Annexe 1]

Au bout de deux semaines, les patients reviendront au centre avec le système **Actimyo** et la même série de tests sera enregistrée.

8.2. Déroulement des évaluations

Dans l’environnement contrôlé les tests sont les suivants :

- Tests de force :
    - *Hand Grip* - main dominante puis non-dominante, 3 essais
    - *Pinch* test - main dominante puis non-dominante, 3 essais

- Tests quantifiés (avec scores)
    - *Box and block* test - main dominante puis non-dominante, 3 essais
    - Minnesota test (variante) - main dominante puis non-dominante, 3 essais
    - Moviplate - main dominante puis non-dominante, 3 essais
    - Taper à l'ordinateur une phrase en continu: « Les pompiers sont partis de la caserne avec leur camion rouge» - main dominante, 3 essais
    - Boutonner une veste – (Dispositif sur la main dominante), 1 essai
- Mouvements libres
    - prendre son repas de midi (Dispositif sur la main dominante)
    - se brosser les dents (Dispositif sur la main dominante)
    - écrire la phrase « Les pompiers sont partis de la caserne avec leur camion rouge» (Dispositif sur la main dominante)

9. DESCRIPTION DES EVALUATIONS

Actimyo (Bere et al. 2011)[19] est un système qui a pour but de mesurer l'activité physique d'un patient à partir de l'extraction des données issues de capteurs fixés sur un membre et sur le fauteuil roulant électrique.

Il est constitué d'un ou plusieurs blocs capteurs, d'un boîtier d'interface capteurs/PC et d'un logiciel d'acquisition/décodage des données installé sur le PC. Chaque bloc est constitué d'un accéléromètre, d'un gyroscope et d'un magnétomètre mesurant respectivement les accélérations, les vitesses angulaires et le champ magnétique dans les 3 directions de l'espace. Ceux-ci sont positionnés sur le membre dont on souhaite étudier le mouvement. Le boîtier d'interface capteurs/PC permet de synchroniser les blocs capteurs et de centraliser les données. Le logiciel permet le décodage des données et leurs récupérations sous forme de fichiers Excel. Toutes les communications Capteurs ↔ Boîtier ↔ PC se font par transmission

---

[19] Elodie Béré, Mark Benoit, David Vissière, Jacques Duchêne, Jean-Yves Hogrel, Laurent Servais, *Actimetry & Neuromuscular Disorders: a system suitable for upper limb evaluation*, Myology 2011

radio Bluetooth. Les capteurs et le boîtier d'interface sont autonomes du point de vue énergétique. Le fonctionnement sera sur batteries rechargeables la nuit.

Le bloc capteur sera protégé dans un boîtier plastique sous forme de montre et porté comme une montre sur le poignet du membre dominant les lundi, mercredi, vendredi et dimanche, et non dominant les mardi, jeudi et samedi. Un autre bloc capteur pourra être placé sur le poignet du membre non dominant. Le boîtier en plastique est suffisamment petit et léger (38 g, 43/33/20 mm) pour ne pas contraindre les mouvements du sujet. [Annexe 2]

Handgrip est un outil qui permet de mesurer la force de préhension palmaire du sujet. Le patient devra serrer le plus fort possible la poignée du Handgrip d'abord avec sa main dominante puis avec sa main non dominante. On réalisera trois essais consécutifs de chaque côté. [Annexe 3]

Pinch test est un outil qui permet de mesurer la force de la pince pouce-index. Le patient devra pincer le plus fort possible l'outil de mesure avec sa main dominante puis sa main non dominante. On réalisera trois essais consécutifs de chaque côté. [Annexe 4]

Box and block test (BBT) est un test de dextérité manuelle, initialement développé pour des adultes atteints de paralysie cérébrale (Mathiowetz et al., 1985)[20],[21]. Le test se compose d'une boîte séparée en deux compartiments égaux, l'un d'eux contient un certain nombre des cubes en bois. Il sera demandé au patient de saisir chaque cube avec une main et de le poser dans le compartiment vide. Le score correspond au nombre de cubes déplacés de l'autre côté pendant 60 secondes. Le test est réalisé à trois reprises, en alternant membre dominant et non dominant. [Annexe 5]

Minnesota test pour la dextérité manuelle est un test standardisé qui permet l'évaluation de la capacité d'un sujet à déplacer des disques à des petites distances. Il est couramment utilisé dans l'évaluation de l'aptitude professionnelle, l'évaluation d'invalidité (Gloss

---

[20] Mathiowetz V, Volland G, Kashman N, Weber K. Adult Norms for the Box and Block Test of Manual Dexterity. Am J Occup Ther 39:386-391, Jun 1985

[21] Mathiowetz V, Federman S, Wiemer D. Box and blocks test of manual dexterity: norms for 6–19 year olds. Can J Occup Ther. 1985;52:241–245.

et al., 1982)[22], et de réadaptation. Nous proposons une variante à 7 disques en bois, colorés sur une face, rangés en « V » inversé. Le patient doit retourner les disques vers la face colorée, en commençant par son côté dominant, puis vers la face non colorée par le côté dominante, plusieurs fois, pendant 60 secondes Le score correspond au nombre de disques retournés. On réalisera trois essais alternativement de chaque côté en commençant par la main dominante. [Annexe 6]

Moviplate est un dispositif développé par l'institut de Myologie et a été validé pour les patients DMD (Servais et al., 2010)[10]. Le dispositif comporte deux plates-formes. Le but est simplement de toucher alternativement chacune d'entre elles. Ces plates-formes sont reliées aux capteurs de force pour mesurer directement la force développée. Un compteur indique le nombre de contacts et un chronomètre décompte le temps sur une durée choisie. Le score correspond au nombre de couples de contact en 30 secondes. On réalisera trois essais alternativement de chaque côté en commençant par la main dominante. [Annexe 7]

Taper une phrase sur le clavier d'ordinateur : est une tache de la vie quotidienne, nous demandons au sujet de taper avec un doigt de la main dominante le plus de fois possible pendant 60 secondes la phrase suivante (sans signe de ponctuation, sans majuscule, sans correction) : « Les pompiers sont partis de la caserne avec leur camion rouge». Le score correspond au nombre de caractères (les espaces compris) tapés. On réalisera trois essais.

Les boutons de la veste : Il sera demandé au patient de boutonner complètement une veste dont les manches auront préalablement été enfilées, pendant 60 secondes. Le score correspond au nombre de boutons boutonnés.

Les mouvements libres : Le patient recevra dans le cadre de ce protocole un repas de son choix (bon repas à la cafétéria). L'enregistrement sera réalisé lors de la consommation de celui-ci. Lors du deuxième enregistrement 15 jours plus tard, il sera demandé au patient de choisir le même repas. Il sera ensuite demandé au patient de se brosser les dents (dentifrice déposé sur la brosse à dent et brosse à dent placée sur le rebord de l'évier, puis brossage pendant 60 secondes). Le patient sera également enregistré pendant une minute par ActiMyo

---

[22] Gloss DS, Wardle MG., *Use of the Minnesota Rate of Manipulation Test for disability evaluation,* Percept Mot Skills. 1982 Oct;55(2):527-32.

pendant la rédaction manuelle de la phrase «Les pompiers sont partis de la caserne avec leur camion rouge» . Toutes ces tâches seront exécutées une seule fois, sans consigne particulière (les patients myopathes présentent de nombreuses stratégies d'adaptation).

L'utilisation d'ActiMyo en dehors de l'Institut de Myologie

Le patient quittera l'Institut de Myologie en emportant le dispositif Actimyo (un boitier placé au poignet et un boitier placé sur le fauteuil). Préalablement une explication complète du fonctionnement lui aura été donnée. À la fin de la journée, Actimyo sera enlevé du poignet et branché au port USB du PC du patient pour se recharger. Le lendemain, avant de commencer ses activités usuelles, Actimyo sera re-attaché au poignet par le patient lui-même ou par quelqu'un de son entourage. Le nouvel enregistrement commence automatiquement. Le boitier attaché au niveau du fauteuil roulant sera mis sous tension (le matin) et hors tension (à la fin du jour) en appuyant sur un seul bouton.

Le patient portera avec lui un petit agenda qui sera fourni par nous pour qu'il puisse noter l'heure et la date du début et de la fin d'une activité (comme se brosser les dents, manger, utiliser l'ordinateur, s'habiller (voir annexe 1).

10. RECUEIL DE DONNEES

Les évaluations seront réalisées par un kinésithérapeute, un psychomotricien ou un médecin entraîné. Une feuille de saisie des données sera utilisée pour noter les résultats des tests ci-dessus. Par ailleurs, un examinateur notera les mouvements passifs (induits par un soignant ou un parent) pendant la journée d'évaluation.

11. BENEFICE ATTENDU

Il n'y a pas de bénéfice attendu pour les patients se prêtant à cette étude.

12. DUREE D'EXCLUSION

Il n'est pas prévu de période d'exclusion après l'inclusion des patients. Il n'y a pas de suivi prolongé, ni d'effets rémanents à prévoir.

13. GESTION DES SORTIES D'ESSAI

Les sujets qui ne voudront pas aller jusqu' au bout du protocole pour une raison ou une autre pourront quitter l'essai sans justification, ils seront remplacés par un autre sujet appartenant à la même sous population étudiée. Les patients qui ne parviennent pas à réaliser une ou plusieurs des taches pourront continuer le protocole.

14. GESTION DES EVENEMENTS INDESIRABLES

Il n'y a pas d'évènements indésirables graves prévisibles pour cette étude étant donné que les tests cliniques prennent place dans un environnement sûr et contrôlé, et que ces tests ne présentent en soi aucun risque. L'ensemble des tests est non invasif et n'expose à aucun effet secondaire sérieux. Les risques associés à cette étude sont ceux liés à la réalisation de mouvements forcés (fatigue, douleurs musculaires, crampes). Les évaluations musculaires seront arrêtées en cas d'apparition de douleur ou de crampe.

Tous les événements indésirables, c'est-à-dire tous les symptômes, signes cliniques, réactions qui se développeraient ou s'aggraveraient lors de l'étude, seront immédiatement enregistrés à la place prévue à cet effet sur la fiche expérimentale et sera diffusée à l'investigateur principal ainsi qu'au promoteur. Les évènements indésirables graves seront déclarés dans les 48h au promoteur. L'évolution de chaque événement sera suivie jusqu'à guérison, stabilisation ou jusqu'à ce qu'il soit clairement établi un commun accord que l'événement n'est pas lié à la recherche en cours.

Pendant l'utilisation de l'appareil à la maison, tous les effets indésirables survenus seront signalés au médecin investigateur. Un numéro de téléphone ainsi qu'une adresse e-mail sera donné au patient et à ses parents pour toute question relative au fonctionnement de l'appareil ou pour signaler tout problème technique.

15. INDEMNISATION

La participation au présent protocole est libre et ne donne pas lieu à une indemnisation. Néanmoins, les frais de déplacement des patients et de leurs parents/représentants légaux seront pris en charge par le promoteur sur base de présentation des justificatifs originaux (transport selon le choix : train, taxi, VSL ….) ou d'une indemnité kilométrique forfaitaire de 0.32 euros/km en cas d'utilisation du véhicule.

Les frais de repas pour les patients et leurs accompagnants seront pris en charge pour un montant maximal de 15 euros par personne.

16. ENGAGEMENT DE L'INVESTIGATEUR

La recherche sera menée dans le respect de la réglementation française en vigueur, notamment des dispositions relatives à la recherche biomédicale du Code le la Santé Publique, article L 1121-1 et suivant, des lois de Bioéthique, de la loi Informatique et Libertés, de la déclaration d'Helsinki, ainsi que des Recommandations de Bonne Pratique et du présent protocole.

Avant la mise en œuvre de la recherche, l'investigateur coordonnateur soumettra le projet à l'avis du CPP Ile-de-France VI (GH Pitié-Salpêtrière - 47, Boulevard de l'Hôpital - 75651 PARIS Cedex 13) en accord avec l'article L 209 de la loi Huriet de 1988 et lui fournira pour cela tous les renseignements nécessaires, formulaire d'information et de consentement ou tout autre document pertinent devant être présenté au Comité. L'essai ne pourra débuter que lorsque l'Institut de Myologie aura été informé de l'avis favorable sans réserve délivré par le CPP à propos du protocole soumis. Le promoteur informera le CPP de tous les évènements indésirables graves ou inattendus et faits nouveaux apparaissant au cours de la recherche et qui affecteraient vraisemblablement la sécurité des personnes s'y prêtant.

L'investigateur s'engage à mener la recherche conformément à ces dispositions éthiques et réglementaires. Il est conscient que tous les documents ainsi que toutes les données relatives à la recherche pourront faire l'objet d'audits et d'inspections réalisées dans le respect du secret professionnel et sans que puisse être opposé le secret médical. L'investigateur reconnaît que les résultats de la recherche sont la propriété de l'Institut de Myologie, promoteur de la recherche.

L'Institut de Myologie, en tant que promoteur a souscrit pour toute la durée de l'essai un contrat d'assurance sous le N°……………, conformément aux dispositions légales et réglementaires françaises sur les recherches biomédicales.

17. LETTRE D'INFORMATION ET CONSENTEMENT ECLAIRE

Le consentement éclairé, signé par les personnes qui se prêtent à la recherche, doit être obtenu par l'investigateur, inscrit à l'ordre des médecins, déclaré en tant qu'investigateur auprès du promoteur, avant tout acte pratiqué dans le cadre du protocole de la recherche et quel que

soit cet acte, conformément à la réglementation. Le consentement ne peut être recueilli qu'après la lecture de la note d'information et après que le patient ait eu l'occasion de poser toutes les questions nécessaires.

Pour les patients mineurs, le consentement des titulaires de l'autorité parentale sera obtenu. Dans le cas ou l'un des deux titulaires de l'autorité parentale ne peut pas se déplacer lors de la visite d'inclusion, son consentement sera obtenu par courrier avant l'inclusion du patient.

Conformément à la loi du 20 décembre 1988 modifiée, l'information donnée oralement et par écrit aux patients comportera des renseignements sur l'objectif de la recherche, sa méthodologie et sa durée, les contraintes et les risques prévisibles, l'avis du CCP, ainsi que sur le droit de refuser de participer à la recherche ou de retirer son consentement à tout moment sans encourir aucune responsabilité. Les patients donneront leur consentement libre, éclairé et écrit en apposant leur signature et la date sur le formulaire de consentement. Ce formulaire de consentement sera rédigé en 2 exemplaires afin que le patient et le médecin investigateur puissent en garder chacun un exemplaire.

L'investigateur devra s'assurer que la personne qui se prête à la recherche aura eu le temps de prendre sa décision librement et aura pu lire et comprendre le formulaire d'information et de consentement.

Le formulaire d'information et de consentement est un document qui aura été approuvé préalablement à la mise en œuvre de la recherche par le CPP, à l'occasion de l'examen du protocole.

18. LA GESTION DES DONNES

18.1. La confidentialité

Les données individuelles resteront confidentielles et anonymes.

Une feuille de saisie des données sera utilisée pour enregistrer chacune des données ci-dessus. Les patients seront identifiés par un numéro de code constitué par leur numéro d'inclusion (01 pour le premier, 02 pour le deuxième …), les trois premières lettres du nom et les deux premières lettres du prénom. Les consentements éclairés seront remis au promoteur dans une enveloppe scellée à la fin de l'étude.

Les données non informatisées, en particulier écrites, seront archivées dans un endroit sécurisé. Les données informatiques seront protégées par un mot de passe.

18.2. L’analyse des donnés

Les mouvements en environnement contrôlé

Pour chaque patient, une correspondance entre les capacités fonctionnelles (les scores) et les accélérations mesurées, ou toute autre mesure susceptible de résumer l’activité du sujet sera réalisée. Nous utiliserons pour ce faire une corrélation linéaire.

La reproductibilité des mesures pour des tests pour lesquels un même patient a obtenu un même résultat sera évaluée par la méthode des ICC (le coefficient de corrélation intra classe)

Par ailleurs, les deux journées d’évaluation permettront d’évaluer la capacité du programme d’analyse à déterminer les mouvements passifs (notés par un des examinateurs).

Les mouvements en environnement libre

Seront analysés pour :

- détection et exclusion des mouvements passifs (en comparaison avec l’agenda)
- définition d’un seuil pour détecter les périodes d’inactivité/ activité
- déterminer le pourcentage des périodes d’inactivité/activité sur une journée
- sur les périodes d’activités :
    - détection des mouvements passifs
    - reconnaissance de tâche particulière à l’aide des enregistrements réalisés en environnement contrôlé (ex : se brosser les dents, manger, s’habiller…).
- réalisation d’un histogramme de distribution des valeurs du module de l’accélération et du module de la vitesse angulaire
- définir la répartition des accélérations en x, y, z pour analyser la proportion des accélérations contra gravitaires
- comparaison des profils d’accélérations entre chaque sujet pour observer la cohérence entre les capacités fonctionnelles mesurées en milieu contrôlé et l’activité mesurée à domicile.
- comparaison inter jours et inter semaine.

18.3. L’archivage des documents

Les investigateurs conserveront un dossier comportant la liste des sujets inclus avec le code correspondant, la copie de tous les feuillets d'expérimentation et l'ensemble de la documentation relative à l'étude conformément à la réglementation européenne, pour une durée de 15 ans après la fin de l'étude.

Après saisie informatique des données, les originaux des documents seront conservés par le promoteur. Les consentements seront placés par l'investigateur dans une enveloppe scellée remise au promoteur à la fin de l'étude.

19. AMENDEMENTS AU PROTOCOL

Ni les investigateurs, ni le promoteur, ne modifieront le protocole sans l'autorisation de l'autre partie. Toute modification substantielle du protocole devra faire l'objet d'un amendement signé par l'investigateur principal et le promoteur. Il sera soumis pour avis au CPP. L'amendement dûment signé et approuvé sera porté à la connaissance et transmis à l'ensemble des collaborateurs de la recherche.

20. DUREE DE L'ETUDE

La durée totale de l'étude sera de 12 mois de juillet 2011 à juillet 2012. La durée d'inclusion des sujets sera d'environ 3 mois (approximativement entre juillet 2011 et septembre 2011) dès l'obtention des accords.

21. RAPPORT FINAL ET PUBLICATIONS

A la fin de l'étude, un rapport sera rédigé par l'investigateur principal avec la collaboration des différents intervenants. Il pourra donner lieu à des communications écrites, orales ou affichées. Conformément au code de la Santé Publique, chacun des investigateurs ou intervenants s'interdit de publier ou de rapporter les résultats de l'étude sans l'accord formel des autres et l'information de promoteur.

22. ANNEXES

22.1. ANNEXE 1

NOM : **PRENOM :** **DATE :**

Durée d'utilisation ACTIMYO :……heures **J( )** **Main : ………**

HEURES	MISE EN PLACE D'ACTIMYO (heure)	ACTIVITÉS DE LA VIE QUOTIDIENNE (toilette, repas, habillage, loisirs…) (heure)	COMMENTAIRES	RETRAIT D'ACTIMYO (heure)
7h-8h				
8h-9h				
9h-10h				
10h-11h				
11h-12h				
12h-13h				
13h-14h				
14h-15h				
15h-16h				
16h-17h				
17h-18h				
18h-19h				
19h-20h				
20h-21h				

22.2. ANNEXE 2

Actimyo®

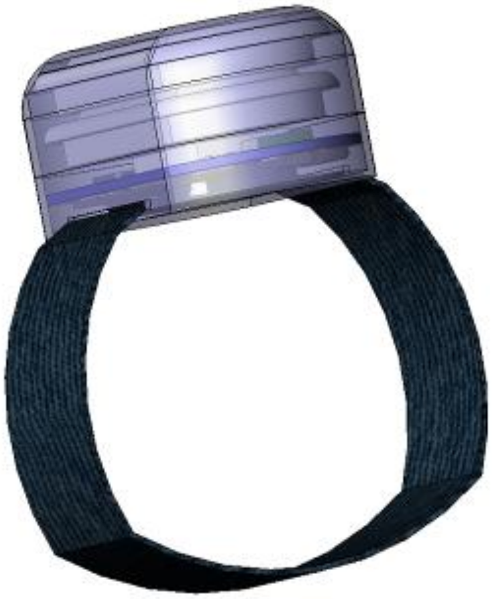

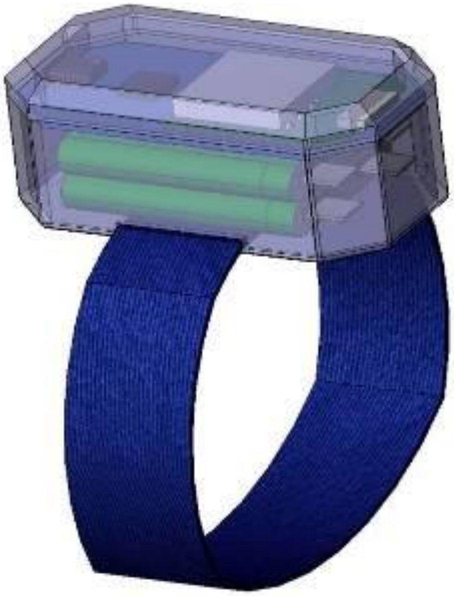

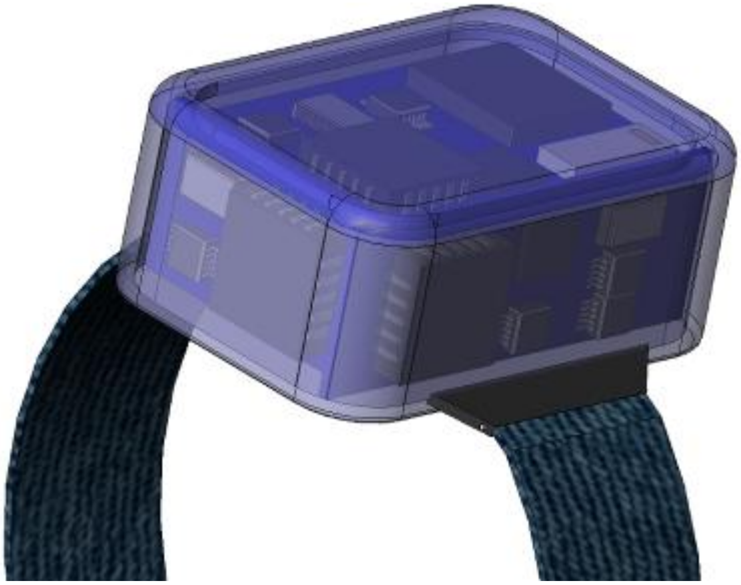

22.3. ANNEXE 3

Hand Grip

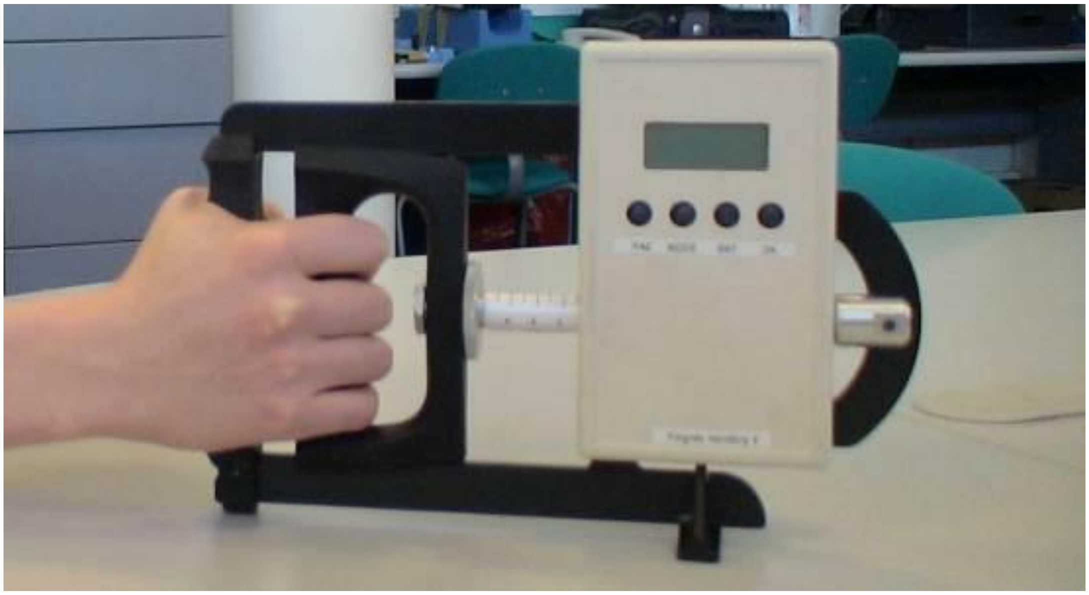

22.4. ANNEXE 4

Pinch

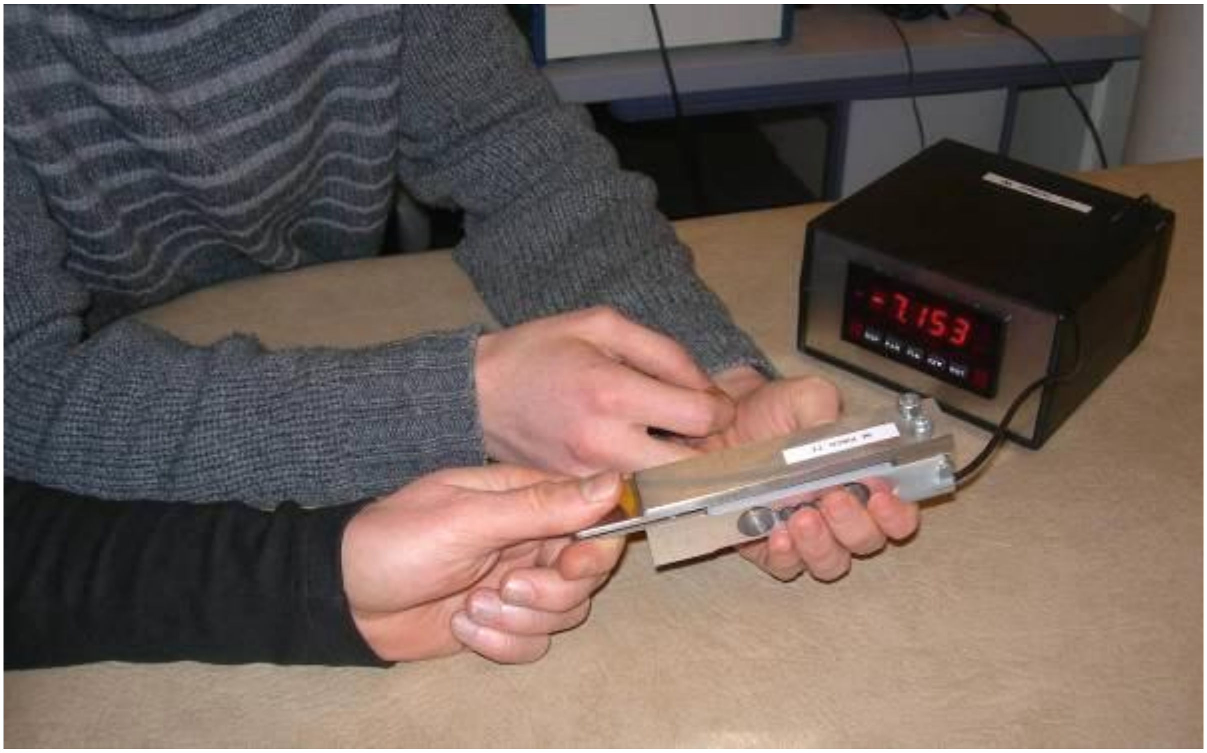

22.5. ANNEXE 5

Block and box test

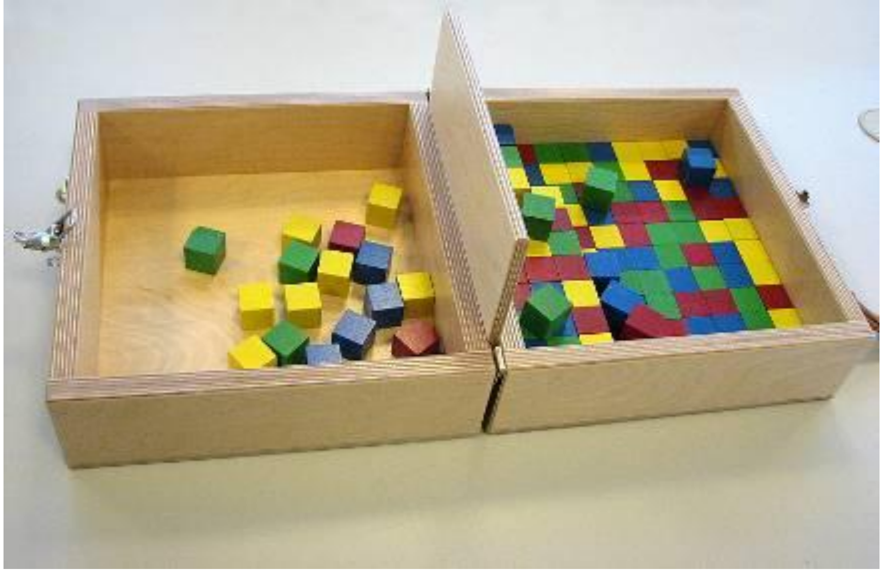

22.6. ANNEXE 6

Minnesota test (7 disques variante)

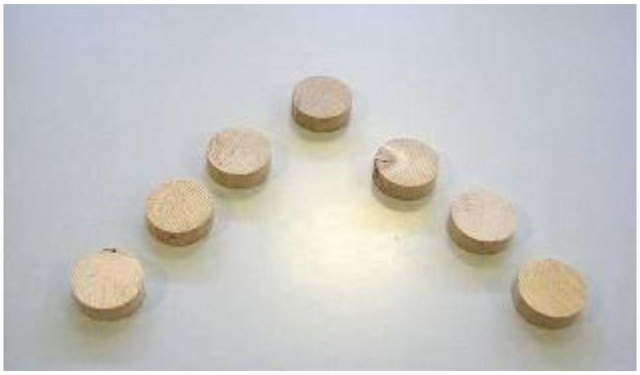

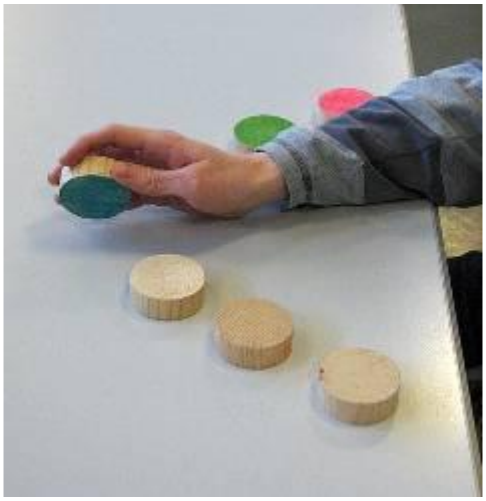

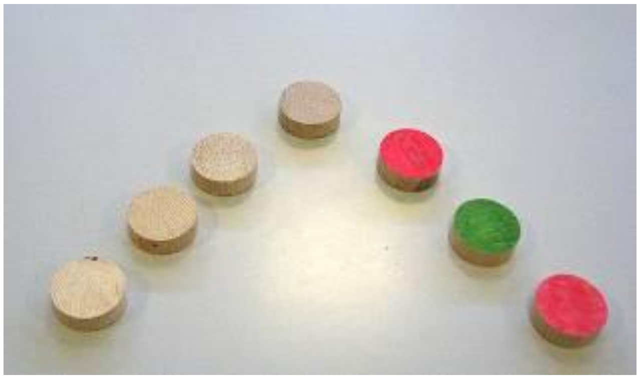

22.7. ANNEXE 7

Moviplate

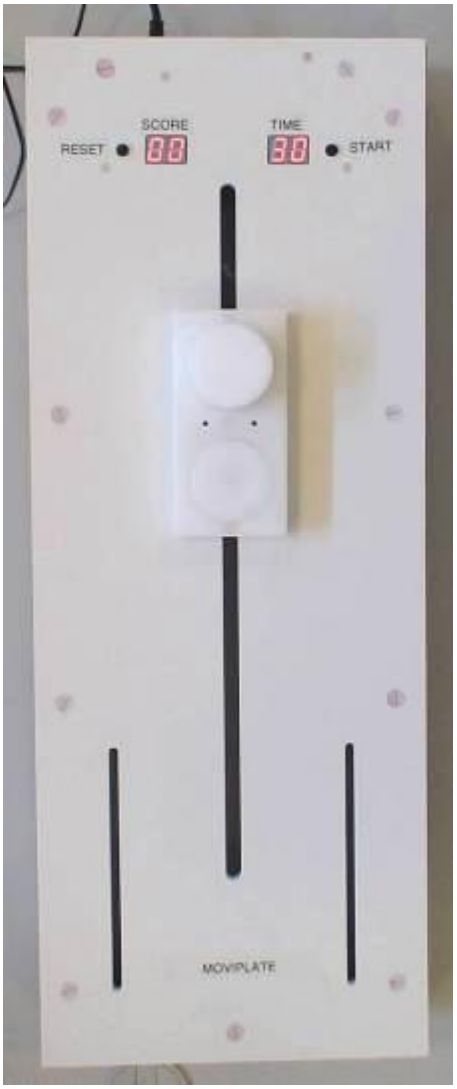

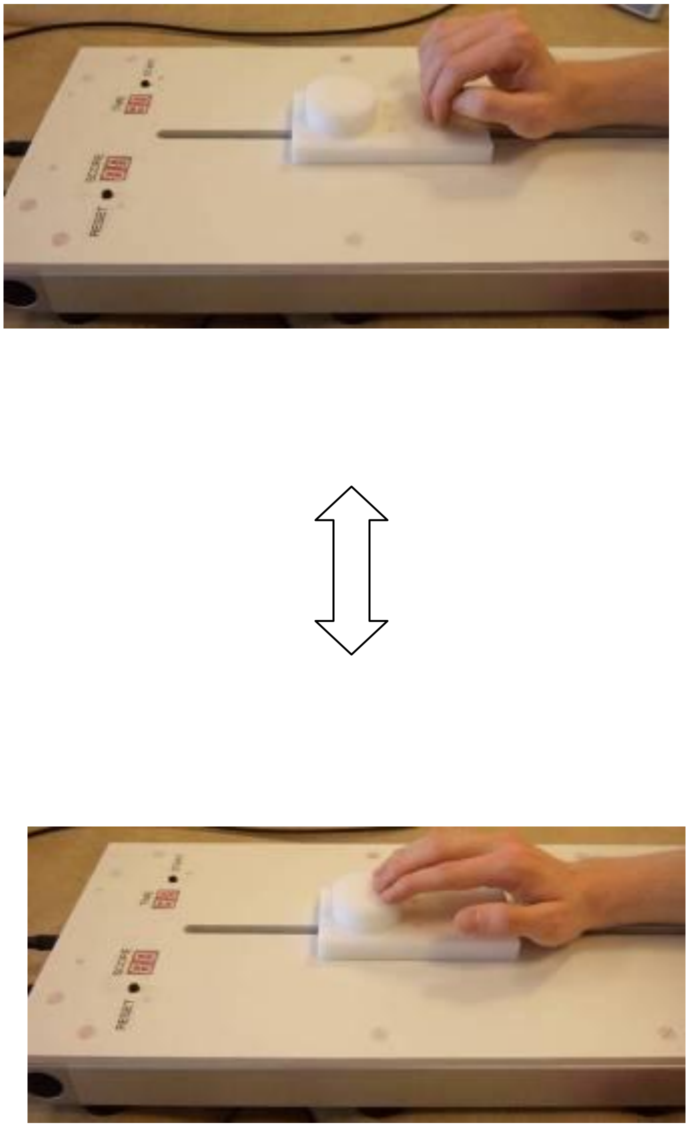

Supplement: S1 Protocol — (PDF) [file pone.0156696.s003.pdf]
