# Supplementary material for: A Movement Monitor Based on Magneto-Inertial Sensors for Non-Ambulant Patients with Duchenne Muscular Dystrophy: A Pilot Study in Controlled Environment
Source: PLoS One. 2016 Jun 7;11(6):e0156696. doi: 10.1371/journal.pone.0156696 (PMC4896626; doi:10.1371/journal.pone.0156696)

PROJECT OF BIOMEDICAL RESEARCH

Continuous activity measure for clinical evaluation at home in non-ambulatory patients with neuromuscular diseases

Pré-Acti Study

Sponsor: Institute of Myology – GH Pitié-Salpêtrière – 75651 PARIS Cedex 13

Tel: 01-42-16-66-46 Fax: 01-42-16-58-87

***Principal investigator*:** Dr. Laurent Servais – Institute of Myology

Tel: 01-42-16-56-70 Fax: 01-42-16-58-56

Co- *investigators*: Anne-Gaëlle LE MOING

Collaborating scientists: Jean Yves HOGREL, Andrea TOTOESCU, Mohamed BENALI, Amélie MORAUX, Aurélie CANAL.

***Experimental plan*:** Evaluation and validation of a measurement system

Type of the study: Multicentre

Places of the study:

- Institute of Myology

- CHU Amiens

Record no. AFSSAPS : 2011-A00795-36

CONTENTS

1. SUMMARY ... 4

2. INTRODUCTION ... 5

3. CONTEXT AND JUSTIFICATION FOR REQUEST ... 7

4. OBJECTIVES ... 9

5. METHODOLOGY ... 9

6. THE INCLUSION CRITERIA ... 9

7. THE EXCLUSION CRITERIA ... 10

8. WORKFLOW TABLE ... 11

8.1. GENERAL COURSE OF THE STUDY ... 12

8.2. PROGRESS OF THE EVALUATION ... 12

9. DESCRIPTION OF THE EVALUATION ... 13

10. DATA COLLECTION ... 16

11. EXPECTED PROFIT ... 16

12. DURATION OF THE EXCLUTION ... 16

13. MANAGEMENT OF WITHDRAWAL FROM A TRIAL ... 16

14. MANAGEMENT OF ADVERSE EFFECTS ... 16

15. COMPENSATION ... 17

16. INVESTIGATOR'S COMMITMENTS ... 17

17. INFORMATION FORM AND INFORMED CONSENT ... 18

18. DATA MANAGEMENT ... 19

18.1. PRIVACY POLICY ... 19

18.2. DATA ANALYSIS ... 19

18.3. Archiving of the documents ... 20

19. AMENDEMENTS TO THE PROTOCOL ... 20

20. DURATION OF THE STUDY 21

21. FINAL REPORT AND PUBLICATIONS 21

22. APPENDIX 22

22.1 Appendix 1 22

22.2 Appendix 2 23

22.3 Appendix 3 24

22.4 Appendix 4 24

22.5 Appendix 5 25

22.6 Appendix 6 25

22.7 Appendix 7 26

1. SUMMARY

The need for specifically adapted measures to assess the strength and residual function of upper limbs in non-ambulatory patients with neuromuscular diseases (particularly Duchenne muscular dystrophy and Spinal muscular atrophy) is still relevant.

Both for a regular monitoring of the clinical evaluation in non-ambulatory patients with neuromuscular diseases and for supplying with a system of quantitative measures for research, the **Pré-Acti** project proposes a continuous and homeevaluation of a complete measuring system of muscular activity in non-ambulant patients.

The principle of the measures consists in the use of the accelerometry and magneto-inertial sensors, an innovative technology invented and developed by the SYSNAV Company., for the evaluation of the linear and angular velocity

The system aims to measure the physical activity of a patient as measured by the sensors in the three dimensional space; data are recorded by the sensors attached to one of his upper limbs.

Ten non-ambulatory patients will be included and assessed at the inclusion and fourteen days later. Patients will wear a small plastic watch-like device during the evaluations and at home for fourteen days.

According to this protocol, we want to demonstrate that the simultaneous recording of linear accelerations and angular velocities in the three axes in non-ambulatory patients with myopathies can be assessed at home in a simple, reliable and reproducible manner and can evaluate the activity of upper limbs. Therefore, the purpose of **ActiMyo** is to provide a primary outcome measure that could be used in clinical trials and clinical follow-up for patients.

2. INTRODUCTION

Duchene muscular dystrophy (DMD) is the most common muscular dystrophy in children. It is a X-linked genetic disease with an incidence of 1/3500 male births (Emery 1991)[1] with a progressive evolution, leading to the loss of walking in the second decade and death in the fourth decade (Kohler et al. 2009)[2]. It affects all the muscles of the body with a progressive restriction of the use of upper limbs. The treatment by glucocorticoids is nowadays the only one considered to have an effect by slowing down the regression of loss of muscle strength and power (Manzur et al., 2008)[3].

The absence of dystrophin in DMD is due to different mutations in the dystrophin gene, disrupting the reading frame. Given the organization in 79 exons of the gene, the reading frame can be restored by the exclusion of one or more exons if the local phase is right. This therapy is called exon skipping (Aartsma-Rus et al., 2002)[4]. These days several trials by exon skipping therapy are already underway or will start soon (Nelson et al., 2009)[5]. A very important aspect is the need to base the clinical trials on reliable and validated measures, that is to say, reproducible, sensitive, and objective.

After the treat-NMD workshop of 11 July 2007, the need for specifically adapted measures to assess the residual strength of upper limbs in non-ambulatory patients was raised (Mercuri et al, 2008)[6]. Therefore, different scales have been proposed, including the Hammersmith Motor

---

[1] Emery, A. E. (1991). "Population frequencies of inherited neuromuscular diseases--a world survey." Neuromuscul Disord **1**(1): 19-29.

[2] Kohler, M., C. F. Clarenbach, et al. (2009). "Disability and survival in Duchenne muscular dystrophy." J Neurol Neurosurg Psychiatry **80**(3): 320-325.

[3] Manzur AY, Kuntzer T, Pike M, Swan A. Glucocorticoid corticosteroids for Duchenne muscular dystrophy. *Cochrane Database Syst Rev* 2008; **1:** CD003725.

[4] Annemieke Aartsma-Rus, Mattie Bremmer-Bout, Anneke A. M. Janson, Johan T. den Dunnen, Gert-Jan B. van Ommen, Judith C. T. van Deutekom, Targeted exon skipping as a potential gene correction therapy for Duchenne muscular dystrophy, Neuromuscular Disorders, Volume 12, Supplement 1, October 2002, Pages S71-S77, ISSN 0960-8966, DOI: 10.1016/S0960-8966(02)00086-X.

[5] Nelson SF, Crosbie RH, Miceli MC, Spencer MJ. , Emerging genetic therapies to treat Duchenne muscular dystrophy. Curr Opin Neurol. 2009 Oct;22(5):532-8.

[6] Mercuri, E., Mayhew, A., Muntoni, F., Messina, S., Straub, V., Van Ommen, G.J., Voit, T., Bertini, E., Bushby, K. (2008). Towards harmonisation of outcome measures for DMD and SMA within TREAT-NMD; report of three expert workshops: TREAT-NMD/ENMC workshop on outcome measures, 12th--13th May 2007, Naarden, The Netherlands; TREAT-NMD workshop on outcome measures in experimental trials for DMD, 30th June--1st July 2007, Naarden,

Scale (HFMS) (Scott et al., 1982)[7], the Egen Klassification Scale (EK) (Steffensen et al., 2001)[8], and the Motor Function Measure (MFM) (Berard et al.2006)[9]. The advantage of these scales is the global evaluation of the functions of upper limbs with a minimum of equipment. However, a real disadvantage is the time required for their application, the dependency on the motivation of the subject and also the subjective dimension of the measure. Another tools are being validated at the Institute of Myology for clinical evaluation of upper limbs in DMD patients. (Servais et al., 2010)[10].

The manual strength measure has recently been proposed as a parameter of disease progression (Mattar et al., 2008)[11].

In addition, a similar problem takes place for other neuromuscular diseases depriving non-ambulatory subjects from participation in clinical trials. This is the case, for example, for exon 51 skipping. The pharmacokinetic trial will only begin for non-ambulatory patients, while these trials are in phase three for ambulatory patients.

Clinical trials for non-ambulatory patients are rather scarce due to the lack of outcome measure tools for these patients.

So far, there is no simple clinical measure adapted for non-ambulatory patients with neuromuscular diseases.

The Netherlands; conjoint Institute of Myology TREAT-NMD meeting on physical activity monitoring in neuromuscular disorders, 11th July 2007, Paris, France. *Neuromuscul. Disord. 18*, 894-903.

7 Scott OM, Hyde SA, Goddard C, Dubowitz V. Quantitation of muscle function in children: a prospective study in Duchenne muscular dystrophy. Muscle Nerve 1982;5:291–301.

8 Steffensen B, Hyde S, Lyager S, Mattsson E. Validity of the EK scale: a functional assessment of non-ambulatory individuals with Duchenne muscular dystrophy or spinal muscular atrophy. Physiother Res Int. 2001;6:119-134.

9 Bérard, C., Payan, C., Fermanian, J., Girardot, F. (2006). A motor function measurement scale for neuromuscular diseases - description and validation study. *Rev. Neurol. (Paris) 162*, 485-493.

10 L. Servais, A. Canal, N. De Coninck, A. Mesdelices, I. Desguerres, S. Quijnao-Roy, S. Mahmoudi, B. Estournet, M. Mayer, C. Themar Noel, T. Voit, J.Y. Hogrel, Upper limb evaluation in non-ambulatory patients with neuromusculardisorders, Neuromuscular Disorders 20 (2010) 596–68

11 Mattar, F.L., Sobreira, C. (2008). Hand weakness in Duchenne muscular dystrophy and its relation to physical disability. *Neuromuscul. Disord. 18*, 193-198.

The actigraphy comprises of activity measurement of a subject during a given period. It can be applied through various approaches (combined or not), including accelerometry. The accelerometry technique records the accelerations undergone by an accelerometer, ideally in three spatial axes. Fixed to the upper limbs of a non-ambulatory patient, the accelerometer allows recording the acceleration of the latter, and thus deducting the strength developed by the subject for mobilizing his upper limbs, with the condition of course to know his orientation, that can be realized thanks to gyroscopes. This non-invasive approach in the follow-up of patients is particularly interesting because it is realized in the subjects' environment that is their home or the place of a ordinary life. The measurement takes place during daily activities; it is thus a more direct reflection of the "real life" of the subjects. Besides, it does not require either the motivation, or the active collaboration of the subject. For DMD patients with intellectual delay and / or attention deficit it is a definite asset.

Pré-Acti is a project developed by the Institute of Myology in association with the SYSNAV Company.

For neuromuscular diseases, the actigraphy by acceleromery is in the trial only for ambulant patients, in order to quantify episodes of walking (Jeannet et al, 2011)[12].

The objective of this study is to develop a tool capable to demonstrate the clinical impact of an intervention in patient's daily life, the essential component of medicine and rehabilitation (Bonato, 2005)[13], and at the same time, to improve the monitoring of the disease progression.

3. CONTEXT AND JUSTIFICATION FOR REQUEST

As we have mentioned, it is essential to define a simple, feasible and reliable clinical measure of the muscular function to evaluate the effect of new clinical approaches for patients with neuromuscular diseases (especially DMD, SMA...).

---

[12] Pierre-Yves Jeannet, Kamiar Aminian, Clemens Bloetzer, Bijan Najafi, Anisoara Paraschiv-Ionescu, Continuous monitoring and quantification of multiple parameters of daily physical activity in ambulatory Duchenne muscular dystrophy patients, European Journal of Paediatric Neurology, Volume 15, Issue 1, January 2011, Pages 40-47

[13] Paolo Bonato, Advances in wearable technology and applications in physical medicine and rehabilitation, Journal of NeuroEngineering and Rehabilitation 2 (2005) 1–4.

The accelerometry has already been applied for the evaluation of the physical activity of the obese children (Tanaka et al., 2007)[14], the elderly (Auvinet et al., 2003)[15] as well as for the evaluation and the monitoring of the people with Parkinson's disease (Giuffrida et al., 2009)[16], (Patel et al., 2007)[17]. The evolution is already sensitive for myopathies, and actigraphy measures have recently made a substantial progress (Servais et al., 2009)[18].

---

[14] Chiaki Tanaka, Shigeho Tanaka, Junko Kawahara, and Taishi Midorikawa, *Triaxial accelerometry for assessment of physical activity in young children.* Obesity, 2007. **15**(5): p. 1233-1241.

[15] Bernard Auvinet, Gilles Berrut, Claude Touzard, Laurent Moutel, Nadine Collet, Denis Chaleil, Eric Barrey, *Gait abnormalities in elderly fallers.* Journal of Aging and Physical Activity, 2003. **11**(1): p. 40-52.

[16] Giuffrida, J.P., et al., *Clinically Deployable Kinesia (TM) Technology for Automated Tremor Assessment.* Movement Disorders, 2009. **24**(5): p. 723-730.

[17] Shyamal Patel, Konrad Lorincz, Richard Hughes, Nancy Huggins, John H. Growdon, Matt Welsh, Paolo Bonato, *Analysis of feature space for monitoring persons with Parkinson's disease with application to a wireless wearable sensor system.* 2007 Annual International Conference of the Ieee Engineering in Medicine and Biology Society, Vols 1-16, 2007: p. 6291-6294.

[18] Servais L, Canal A, Hogrel JY, Voit T, *Quantification of upper limb activity in non ambulant boys with Duchenne muscular dystrophy using accelerometry: A proof-of-concept study.* Neuromuscular Disorders, 2009. **19**(8-9): p. 602-603.

4. OBJECTIVES

Pré-Acti is a study of validation. It consists of highlighting the relevance of a clinical measure at home using ActiMyo for non-ambulant patients with neuromuscular diseases.

This protocol aims to demonstrate the following:

1) the feasibility

2) the reliability

3) the validation of the recorded data by ActiMyo.

Therefore, the objective of this study is to envisage a new clinical measure adapted for non-ambulatory patients with neuromuscular diseases.

5. METHODOLOGY

We plan to include 10 non-ambulatory patients with neuromuscular diseases (DMD, SMA) older than 10 years old. The non-ambulatory patients followed at the Institute of Myology and in the Pediatric Neurology department of the CHU of Amiens will be requested to participate in this study. They will be selected by Doctor Laurent SERVAIS at the Institute of Myology and by Doctor Anne-Gaëlle Le MOING at the Hospital of Amiens.

Muscle evaluations will be conducted in the laboratory of Neuromuscular Physiology, headed by Jean-Yves Hogrel at the Institute of Myology. All the tests will be conducted for neuromuscular patients during a scheduled visit to the Institute of Myology. Patients who want to participate in the study will receive a copy of the information form. The reading of it will be organized during a meeting with an investigator. Once this reading is done and the consent is signed, patients can be included in the study.

6. THE INCLUSION CRITERIA

The patients have to satisfy the following criteria:

- older than ten years old
- confirmed diagnosis of a neuromuscular disease (DMD, SMA) by the genetics or by the muscular biopsy
- not able to walk ten meters without outside human help or cane
- able to sit on a wheelchair during at least three hours
- assigned to a social security system
- signed informed consent

7. THE EXCLUSION CRITERIA

- Severe cognitive disorders which limit the understanding of the performed tasks
- Acute neurological, inflammatory, infectious, endocrine or orthopaedic pathologies in the last month before the inclusion.
- Scheduled surgery in three weeks after the inclusion
- Upper-limb surgery three months before the inclusion
- Pregnant or breast-feeding women

8. WORKFLOW TABLE

Table 1. Events Calendar

		D0	D0–D14	D14
Environment		controlled	free	controlled
**Collection of medical data**		x		
**Intercurrent events**				x
**Strength tests**	*Hand Grip* test	x		x
	Pinch Test	x		x
**Standardized tasks (scores)** + ActiMyo	*Box and block* test	x		x
	Minnesota test (variant)	x		x
	Moviplate	x		x
**Tests of a daily life (scores)** + ActiMyo	Typing a phrase on the computer	x		x
**Free movements** + ActiMyo	Buttoning up a jacket	x		x
	Having lunch	x		x
	Brushing the teeth	x		x
	Writing a phrase	x		x
ActiMyo continuously (approximately 15h/d) + Diary			x	

8.1. GENERAL COURSE OF THE STUDY

During their first visit, the patients will meet a medical investigator who will give them an information form for reading and will answer their questions. The informed consent will then be collected and medical data will be written down (weight, size, pathology, Brooke and Walton score, and medical-surgical history). The patients will receive all the necessary information regarding the use of the **Actimyo** device (see below). The patients will be then asked to perform series of tasks in upper limbs, the accelerations and angular velocities will be recorded by the **Actimyo** device. All the tests take about three hours. This first phase is intended to correlate the measurements with the results validated in controlled environment.

Thereafter, the patients will be trained to wear the Actimyo device for two next weeks. The recordings will be only during the day, the batteries of the device must be recharged every night. The patient will be asked to remove the device when having a bath in order to avoid immersion. In all, about fifteen hours of usual activity will be recorded daily in subject's environment. Throughout this period, the patient will maintain a diary of his daily activities, making notes by themselves or by a close relative, the time of the beginning and the end of the activity (for example brushing teeth, eating, using the computer, and dressing). [Appendix 1]

In two weeks, the patients will return to the centre with the **Actimyo** device and the same series of tests will be recorded.

8.2. PROGRESS OF THE EVALUATION

In the controlled environment, there are the following tests:

- Tests of strength
    - *Hand Grip* – dominant then non-dominant hand, three trials
    - *Pinch* test - dominant then non-dominant hand, three trials
- Quantified tests (with scores)

- *Box and block* test - dominant then non-dominant hand, three trials
- Minnesota test (variant) - dominant then non-dominant hand, three trials
- Moviplate - dominant then non-dominant hand, three trials
- Continuous typing a phrase on the computer: "*Les pompiers sont partis de la caserne avec leur camion rouge*" ("The fire brigade left the fire station with their red truck") - dominant hand, three tries
- Buttoning up a jacket – (device on the dominant hand), one try

• Free movements

- Having lunch (device on the dominant hand)
- Brush the teeth (device on the dominant hand)
- Writing the phrase (device on the dominant hand) "*Les pompiers sont partis de la caserne avec leur camion rouge*" ("The fire brigade left the fire station with their red truck ")

9. DESCRIPTION OF THE EVALUATION

Actimyo (Bere et al. 2011)[19] is a system which aims to measure the physical activity of a patient from the extraction of the data recorded by sensors fixed on the limb and on the electric wheelchair.

It consists of one or more sensor blocks, a sensor/PC interface (wheelchair box) and the software of data acquisition/decoding installed on the PC. Each block consists of an accelerometer, a gyroscope, and a magnetometer, measuring respectively the acceleration and the angular speed in the magnetic field in three spatial axes. It is fixed on the limb for which we plan to study the movement. The sensor/PC interface synchronizes with sensor blocks and can centralize the data. The software can decode the data and recover them as Excel files. All

[19] Elodie Béré, Mark Benoit, David Vissière, Jacques Duchêne, Jean-Yves Hogrel, Laurent Servais, *Actimetry & Neuromuscular Disorders: a system suitable for upper limb evaluation*, Myology 2011

connections Sensors ↔ Interface ↔ PC are through Bluetooth radio transmission. The sensors and the interface box are autonomous from the point of view of energy. The functioning will be based on rechargeable batteries at night.

The sensor block will be protected by a plastic box as shown and worn as a watch on the wrist of the dominant limb on Monday, Wednesday, Friday and Sunday, and on the non-dominant limb on Tuesday, Thursday and Saturday. Another sensor block may be placed on the wrist of the non-dominant limb. The plastic box is small and light enough (38 g, 43/33/20 mm) not to intervene with the subject's movement. [Appendix 2]

Handgrip is a tool to measure the strength of the palmar grip of the subject. The patient will grasp as hard as possible the handle of Handgrip, first with his dominant and then with his non-dominant limb. Three consecutive trials will be done on each side. [Appendix 3]

Pinch test is a tool to measure the strength of the thumb-index pinch. The patient will pinch the measurement tool as strongly as possible with his dominant and non-dominant limb. Three consecutive tries will be realized on each side. [Appendix 4]

Box and Block Test (BBT) is a manual dexterity test, originally developed for adults with cerebral palsy (Mathiowetz et al., 1985). The test consists of a box separated on two equal compartments; one of them contains a number of wooden blocks. The patient will be asked to grab each block with one hand and put it into the empty compartment. The score corresponds the number of blocks moved to the other side during sixty seconds. The test is performed three times, alternating dominant and non-dominant limb. [Appendix 5]

Minnesota manual dexterity test is a standardized test, which can assess the ability of the subject to move discs for small distances. It is commonly used for the evaluation of professional competence, for the disability assessment (Gloss et al., 1982), and rehabilitation. We propose seven various wooden discs, coloured on one side, arranged in inverted "V". The patients have to turn the discs on the coloured side, starting with his dominant side, then on the uncoloured side for sixty seconds. The score corresponds the number of returned discs. Three tries will be realized alternately on each side starting with the dominant hand. [Appendix 6]

Moviplate is a device developed by the Institute of Myology and has been validated for DMD patients (Servais et al., 2010). The device includes two platforms. The aim is simply to touch alternately each platform. These platforms are connected to the force sensor, which directly measures the developed strength. A counter indicates the number of contacts and a chronometer

counts the time on a selected duration. The score corresponds the number of pair contacts for thirty seconds. Three tries will be realized alternately on each side starting with the dominant hand. [Appendix 7].

Typing a sentence on the computer keyboard is a task of daily life. We ask a subject to type with one finger of the dominant hand as many times as possible for sixty seconds the following sentence (no punctuation, no capital letters, and no corrections): "*Les pompiers sont partis de la caserne avec leur camion rouge"* ("The fire brigade left the fire station with their red truck."). The score corresponds the number of characters (including spaces) typed. The test will be done tree times.

The buttons of the jacket: The patient will be asked to button up a jacket, the sleeves of which have been previously strung, for sixty seconds. The score corresponds the number of buttoned buttons.

Free movements: According to this protocol, the patient will receive a meal of his choice (a meal ticket in the cafeteria). The recording will be made during the consumption of it. During the second recording, fifteen days later, the patient will be asked to choose the same meal. The patient will also be asked to brush the teeth (toothpaste lying on the toothbrush and toothbrush placed on the edge of the sink and brush for sixty seconds). The patient will also be recorded for a minute by ActiMyo during manual writing of the sentence "*Les pompiers sont partis de la caserne avec leur camion rouge"* («The fire brigade left the fire station with their red truck."). All these tasks will be fulfilled once, without specific instruction, (patients with muscular dystrophy have many adaptation strategies).

Using ActiMyo outside the Institute of Myology

The patient will leave the Institute of Myology, taking the Actimyo device (a sensor placed on the wrist and the otherone placed on the weelchair). Beforehand he will receive a full explanation of its functioning. At the end of the day, Actimyo will be removed from the wrist and will be connected to USB port of patient's PC for charging. The next day, before starting his usual activities, Actimyo will be fasten to the wrist by the patient himself or by someone from his family. The new recording begins automatically. The box fastened to the wheelchair will be switched on (in the morning) and off (at the end of the day) by pressing a button. The patient will have a little diary that will be given to him where he will be able to write down the time and the date of the

beginning and the end of an activity (like brushing his teeth, eating, using the computer, getting dressed (see Appendix 1).

10. DATA COLLECTION

The evaluations will be conducted by a physiotherapist, a psychomotor therapist or a trained medical doctor. An information form will be used to write down the results of the tests mentioned above. In addition, an examiner will write down the passive movements (induced by a doctor or a parent) during an assessment day.

11. EXPECTED PROFIT

Profit is not expected out of the patient participating in this protocol.

12. DURATION OF THE EXCLUTION

The exclusion period is not expected after the inclusion of patients. Neither extended follow-up, nor persistent effect are expected.

13. MANAGEMENT OF WITHDRAWAL FROM A TRIAL

Subjects who do not want to follow the protocol for one reason or another can leave the trial without justification. They will be replaced by another person of similar age. The patients who do not succeed to realize one or more tasks can continue the protocol.

14. MANAGEMENT OF ADVERSE EFFECTS

There are no serious predictable adverse effects for this study given that the clinical tests take place in controlled and reliable environment, and given that these tests present any risk. All the tests are not invasive and do not have any serious side effect. The risks associated with this study, are connected to the realization of forced movements (fatigue, muscular pains, cramps). The muscular evaluations will be stopped in case of appearance of pain or cramp.

All the adverse effects, that is to say all the symptoms, clinical signs, reactions that will develop or will aggravate during the study, will be immediately written in the space provided for

this purpose in the experimental form and the principal investigator as well as to the sponsor informed. The adverse effects will be declared within 48 hours to the sponsor. The evolution of each effect will be followed until recovery, stabilization, or until it is clearly established by mutual agreement that the effect is not related to the current study.

During the use of the device at home, all the adverse effects will be indicated to the medical investigator. A phone number as well as an e-mail address will be given to the patient and to his parents for any question relative to the functioning of the device or for indicating any technical problem.

15. COMPENSATION

The participation in the present protocol is free and does not give any compensation. Nevertheless, the traveling expenses of the patients and their legal parents / representatives will be paid off by the promoter on basis of presentation of the original documentary evidences (transport according to the choice: train, taxi, VSL.) or of a fixed 0.32-euro / km allowance in case of the use of a vehicle.

Meal expenses of the patients and their accompanying person will be paid off a maximum of 15-euro per person.

16. INVESTIGATOR'S COMMITMENTS

The research will be conducted in compliance with the French regulations, including dispositions related to biomedical research in the Public Health Code, Article L. 1121-1 and following, the Bioethics law, the information technology and liberties law (CNIL), the Helsinki Declaration as well as the Recommendations for Good Practice and the present protocol.

Before the implementation of the research, the coordinating investigator will put forward the project to the opinion of the Ethics Committee (CPP) Ile-de-France VI (Pitié-Salpêtrière - 47, Boulevard de l'Hôpital - 75651 PARIS Cedex 13) according to the Article L 209 of the Huriet law and will provide them with all the necessary information, the information form and the consent form or other relevant document before being presented to the Committee. The trial will only begin when the Institute of Myology has been informed of a favorable unreserved opinion issued by the CPP in regards to the protocol submitted. The promoter will inform the CPP about all the serious

or unexpected adverse effects and appearance of new facts that could presumably affect people's safety.

The investigator agrees to conduct the research according to ethical norms and regulations. He is aware that all the documents as well as all the data related to the research may be the subject of audits and inspections in respect of professional confidentiality and may not be confronted with medical secrecy. The investigator recognizes that the study results are the property of the Institute of Myology, sponsor of the research.

The Institute of Myology, as the promoter, subscribed for the entire duration of the trail to an insurance contract №. .... according to French laws and regulations on biomedical research.

17. INFORMATION FORM AND INFORMED CONSENT

The written informed consent of people taking part in the research must be obtained by the investigator, members of the Order of Physicians, declared as an investigator to the sponsor before any act is performed on the basis of the research protocol, accordingly to the regulations. The consent can only be collected after reading of the information form and after the patient had the opportunity to ask all the necessary questions.

For the minor patients, the consent of the holders of the parental rights will be obtained. In the case when one of holders of the parental rights cannot attend the visit of inclusion, his/her consent will be obtained by mail before the inclusion of the patient.

According to the law of December 20th, 1988 as amended, the information given orally and in writing to patients will include information about the purpose of research, methodology and its duration, the constraints and the foreseeable risks, the opinion of the CCP, as well as the right to refuse to participate in the research or to withdraw the consent at any time without any obligation.

Patients will give their free and informed consent in writing by signing and dating the consent form. This consent form will be prepared in two copies for the patient and for the physician-investigator so that each one keeps a copy.

The investigator must ensure that the person suitable for the research will have time to make a decision freely and will be able to read and understand the information form and the consent form.

The information form of consent is a document that will be approved before the implementation of the research by the CPP, after examining the protocol.

18. DATA MANAGEMENT

18.1. PRIVACY POLICY

Personal data will remain confidential and anonymous.

The form for collected data will be used to register all the data. The patients will be identified by a number of the code consisted of their number of inclusion (01 for the first one, 02 for the second), the first three letters of the surname and the first two letters of the name. The informed consents will be returned to the sponsor in a sealed envelope at the end of the study.

The non-computerized data, especially written, will be archived in a secure place. The computerized data will be protected by a password.

18.2. DATA ANALYSIS

<u>The movements in the controlled environment</u>

For every patient, the connection between the functional abilities (the scores) and the measured accelerations, or any other susceptible measurements to summarize the activity of the subject will be used. We will use to make a linear correlation.

The reliability of the measurements of the tests according to which each patient got the same result will be estimated according to ICC method (the correlation of intraclass correlation coefficient).

Besides, two- days' evaluation will allow assessing the ability of the program analysis to determine the passive movements (noted by one of the examiners).

<u>The movements in the free environment:</u>

Will be analysed for:

    - detection and exclusion of the passive movements (in comparison with the diary)
    - definition of a threshold to detect the periods of inactivity / activity
    - determination of the percentage of inactivity / activity periods during the day
    - activity periods:
        - detection of the passive movements
        - recognition of a particular task with the help of recordings in the controlled environment (ex: teeth brushing, eating, getting dressed).
- realization of a histogram of distribution of the module values of the acceleration and the module of the angular speed
- definition of the distribution of the accelerations x, y, z to analyse the proportion of the contra gravity acceleration
- comparison of acceleration profiles between every subject to observe the coherence between the functional abilities measured in the controlled environment and the measured activity at home.
- comparison within a days and within a week.

18.3. Archiving of the documents

The investigators will keep a file containing a list of the included subjects with a corresponding code, the copy of all the papers of the trial and all the documentation related to the study according to European regulations, for a period of fifteen years after the end of the study.

After the data is stored digitally, the original documents will be kept by the promoter. The consents will be returned in sealed envelopes to the promoter by the investigator at the end of the study.

19. AMENDEMENTS TO THE PROTOCOL

Neither the investigators nor the promoter will change the protocol without the consent of the second party.

Any substantial changes in the protocol should be the subject of an amendment signed by the principal investigator and the promoter. It will be submitted by the CPP. The amendment signed and approved will be acknowledged and spread to all the collaborators of the research.

20. DURATION OF THE STUDY

The total study duration will take twelve months from July 2011 to July 2012. The duration of subjects' inclusion will approximately take three months (approximately between July 2011 and September 2011) according to the reached agreements.

21. FINAL REPORT AND PUBLICATIONS

At the end of the study, a report will be prepared by the principal investigator in a collaboration with various participants. It may result in written, oral or poster presentations. According to the Public Health Code, each investigator or participant cannot publish or report the results of the study without the formal agreement with others and informing the promoter.

22. APPENDIX

22.1 Appendix 1

NOM : **PRENOM :** **DATE :**

Durée d'utilisation ACTIMYO :.....heures **J( )** **Main : ....**

HEURES	MISE EN PLACE D'ACTIMYO (heure)	ACTIVITÉS DE LA VIE QUOTIDIENNE (toilette, repas, habillage, loisirs...) (heure)	COMMENTAIRES	RETRAIT D'ACTIMYO (heure)
7h-8h				
8h-9h				
9h-10h				
10h-11h				
11h-12h				
12h-13h				
13h-14h				
14h-15h				
15h-16h				
16h-17h				
17h-18h				
18h-19h				
19h-20h				
20h-21h				
21h-22h				

22.2 Appendix 2

Actimyo®

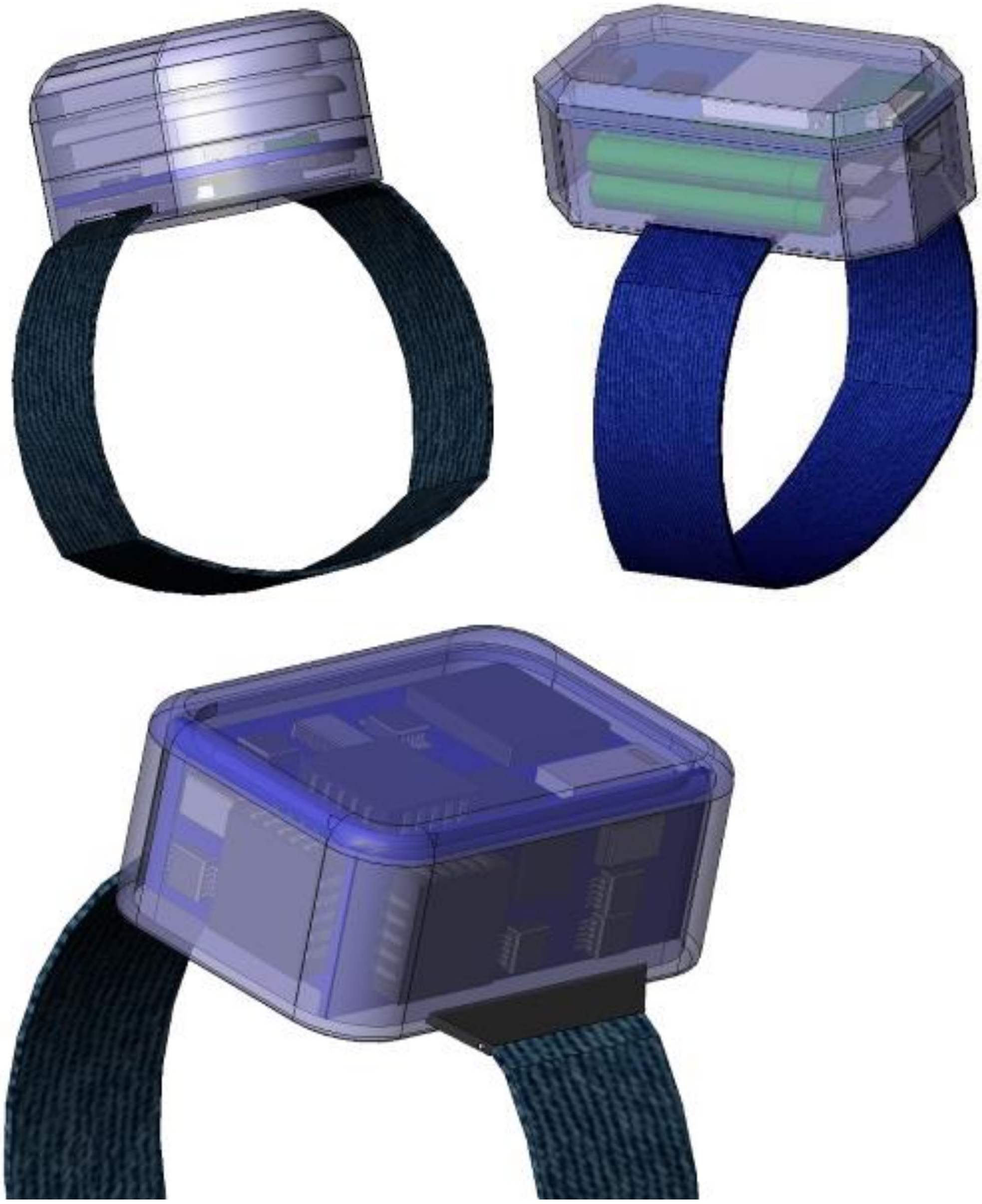

22.3 Appendix 3

Hand Grip

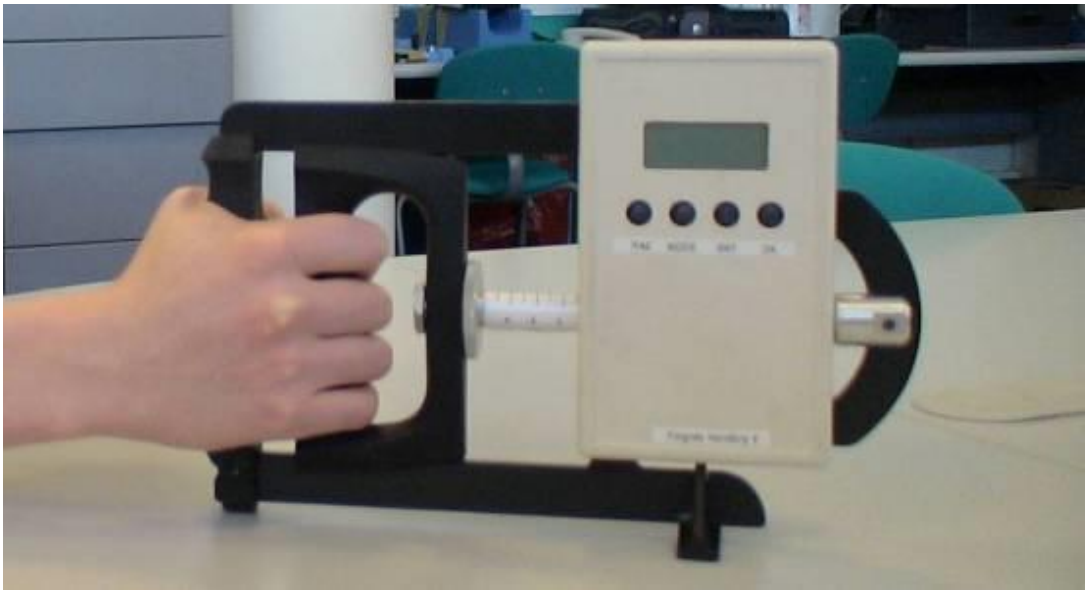

22.4 Appendix 4

Pinch

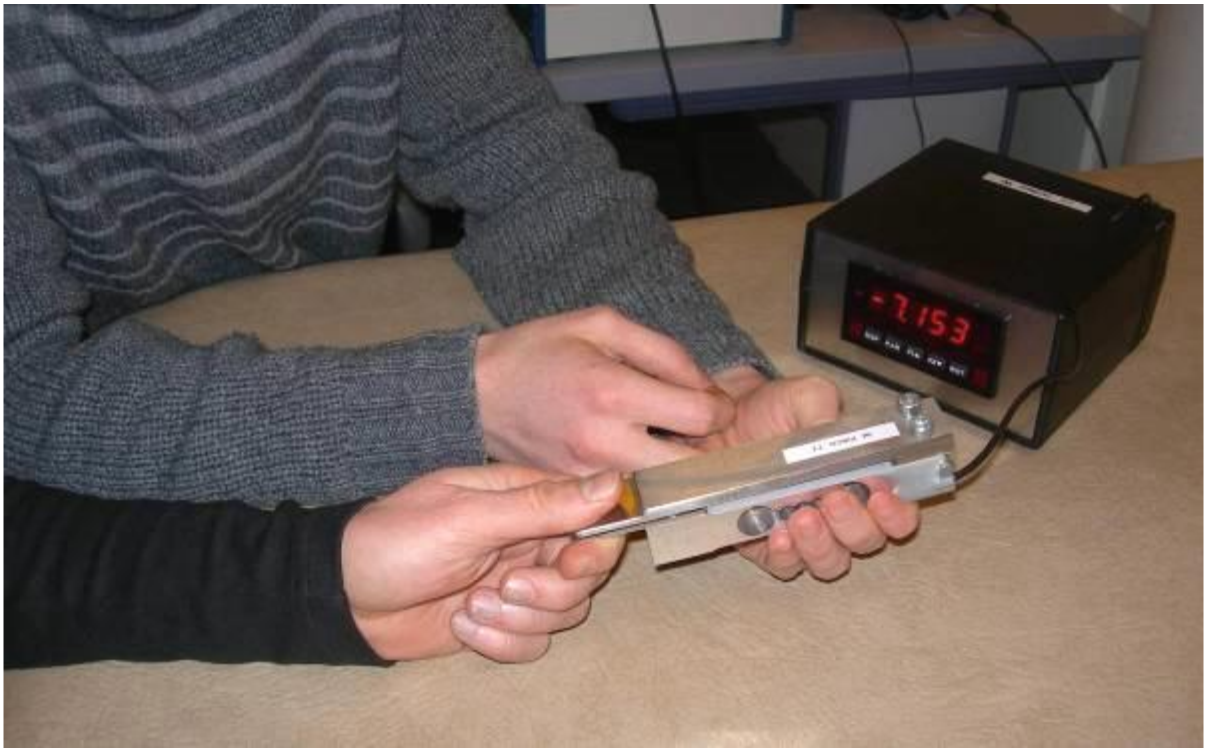

22.5 Appendix 5

Block and box test

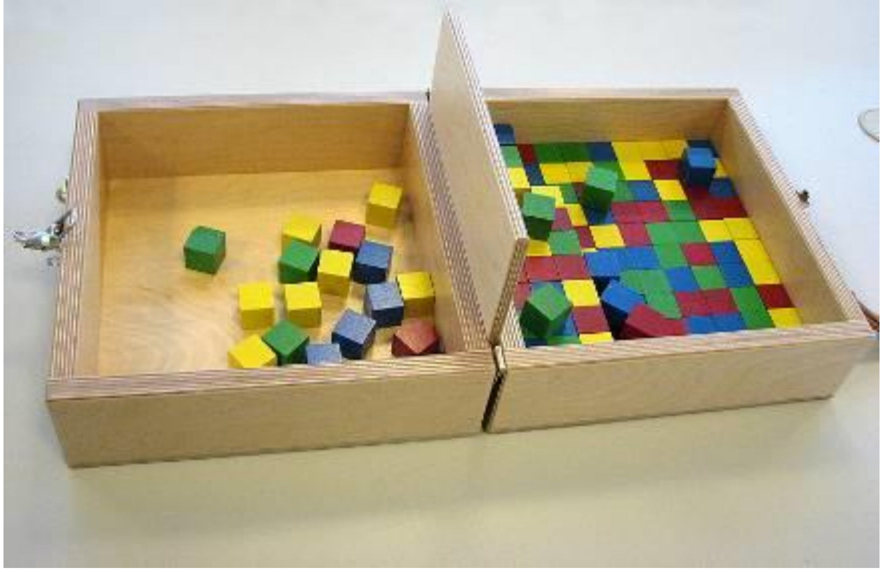

22.6 Appendix 6

Minnesota test (7 discs variant)

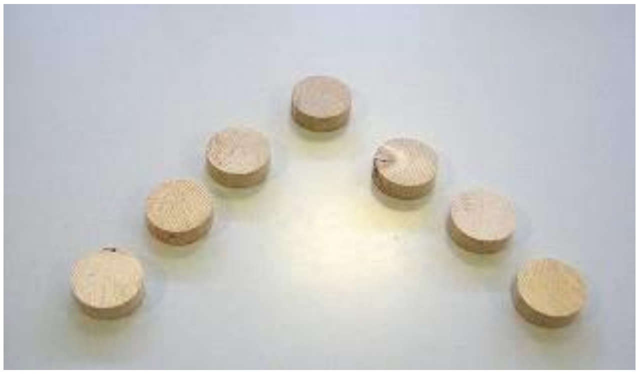

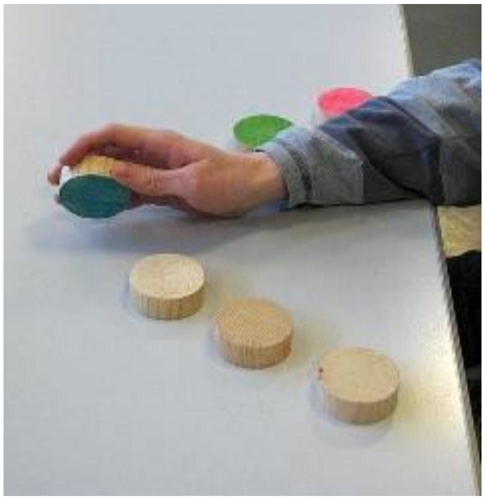

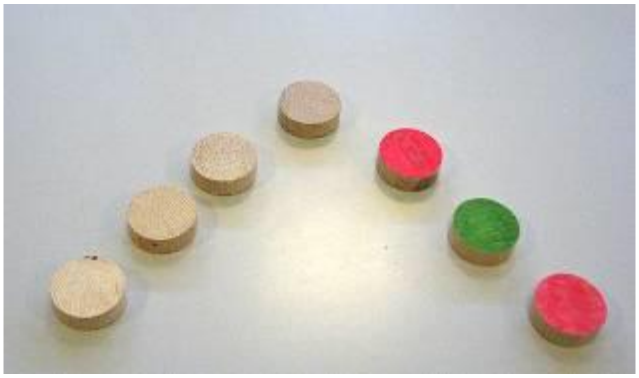

22.7 Appendix 7

Moviplate

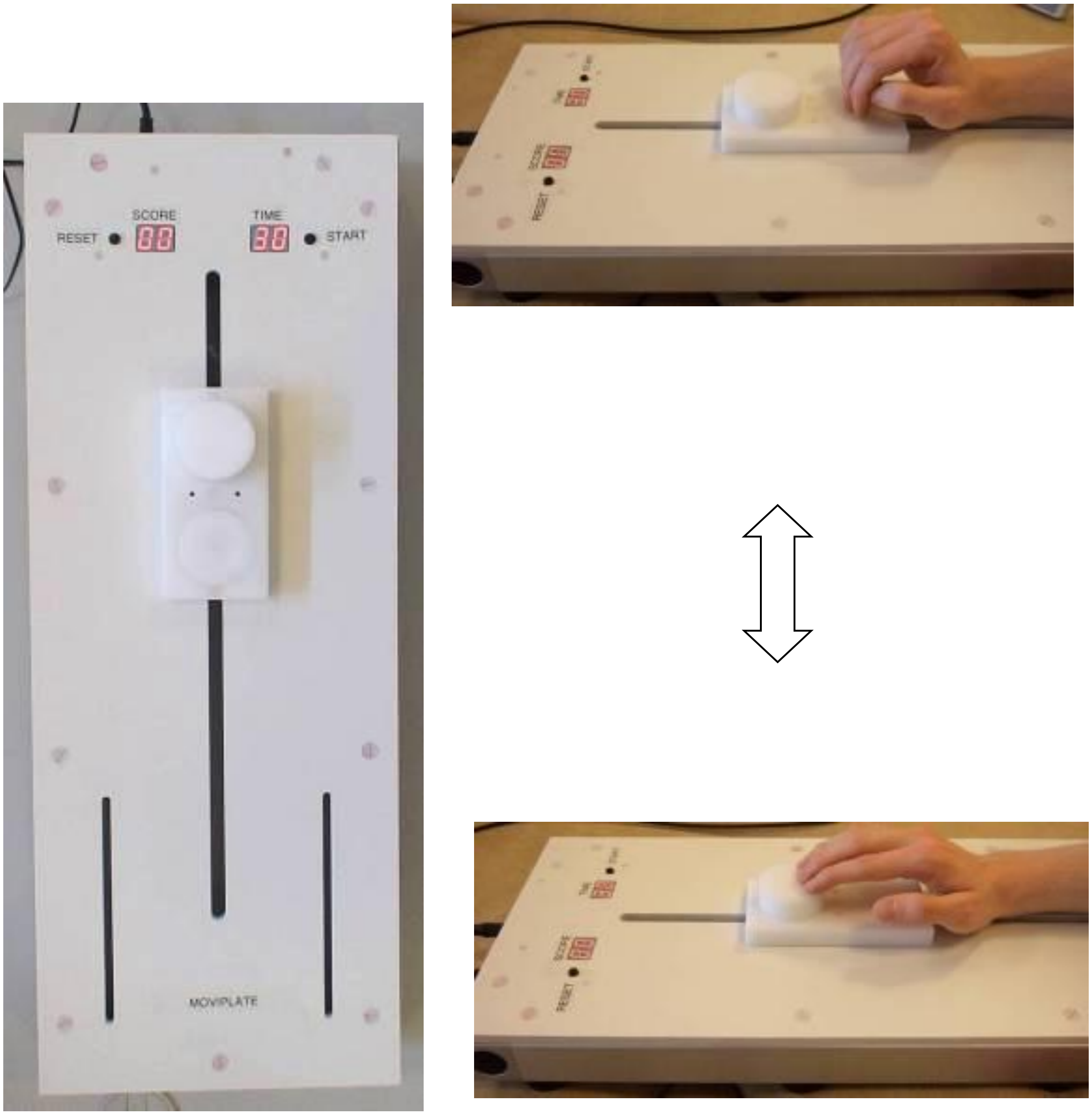

Supplement: S2 Protocol — (PDF) [file pone.0156696.s004.pdf]
